# A SHH-FOXF1-BMP4 signaling axis regulating growth and differentiation of epithelial and mesenchymal tissues in ureter development

**DOI:** 10.1371/journal.pgen.1006951

**Published:** 2017-08-10

**Authors:** Tobias Bohnenpoll, Anna B. Wittern, Tamrat M. Mamo, Anna-Carina Weiss, Carsten Rudat, Marc-Jens Kleppa, Karin Schuster-Gossler, Irina Wojahn, Timo H.-W. Lüdtke, Mark-Oliver Trowe, Andreas Kispert

**Affiliations:** Institut für Molekularbiologie, Medizinische Hochschule Hannover, Hannover, Germany; University of California San Francisco, UNITED STATES

## Abstract

The differentiated cell types of the epithelial and mesenchymal tissue compartments of the mature ureter of the mouse arise in a precise temporal and spatial sequence from uncommitted precursor cells of the distal ureteric bud epithelium and its surrounding mesenchyme. Previous genetic efforts identified a member of the Hedgehog (HH) family of secreted proteins, Sonic hedgehog (SHH) as a crucial epithelial signal for growth and differentiation of the ureteric mesenchyme. Here, we used conditional loss- and gain-of-function experiments of the unique HH signal transducer Smoothened (SMO) to further characterize the cellular functions and unravel the effector genes of HH signaling in ureter development. We showed that HH signaling is not only required for proliferation and SMC differentiation of cells of the inner mesenchymal region but also for survival of cells of the outer mesenchymal region, and for epithelial proliferation and differentiation. We identified the Forkhead transcription factor gene *Foxf1* as a target of HH signaling in the ureteric mesenchyme. Expression of a repressor version of FOXF1 in this tissue completely recapitulated the mesenchymal and epithelial proliferation and differentiation defects associated with loss of HH signaling while re-expression of a wildtype version of FOXF1 in the inner mesenchymal layer restored these cellular programs when HH signaling was inhibited. We further showed that expression of *Bmp4* in the ureteric mesenchyme depends on HH signaling and *Foxf1*, and that exogenous BMP4 rescued cell proliferation and epithelial differentiation in ureters with abrogated HH signaling or FOXF1 function. We conclude that SHH uses a FOXF1-BMP4 module to coordinate the cellular programs for ureter elongation and differentiation, and suggest that deregulation of this signaling axis occurs in human congenital anomalies of the kidney and urinary tract (CAKUT).

## Author summary

The mammalian ureter is a simple tube with a specialized multi-layered epithelium, the urothelium, and a surrounding coat of fibroblasts and peristaltically active smooth muscle cells. Besides its important function in urinary drainage, the ureter represents a simple model system to study epithelial and mesenchymal tissue interactions in organ development. The differentiated cell types of the ureter coordinately arise from precursor cells of the distal ureteric bud and its surrounding mesenchyme. How their survival, growth and differentiation is regulated and coordinated within and between the epithelial and mesenchymal tissue compartments is largely unknown. Previous work identified Sonic hedgehog (SHH) as a crucial epithelial signal for growth and differentiation of the ureteric mesenchyme, but the entirety of the cellular functions and the molecular mediators of its mesenchymal signaling pathway have remained obscure. Here we showed that epithelial SHH acts in a paracrine fashion onto the ureteric mesenchyme to activate a FOXF1-BMP4 regulatory module that directs growth and differentiation of both ureteric tissue compartments. HH signaling additionally acts in outer mesenchymal cells as a survival factor. Thus, SHH is an epithelial signal that coordinates various cellular programs in early ureter development.

## Introduction

The ureter is a pivotal component of the urinary system by warranting the efficient removal of the urine from the renal pelvis to the bladder. This task is accomplished by the compartmentalized organization of the straight tube into an outer flexible but rigid peristaltically active mesenchymal coat with contractile smooth muscle cells (SMCs) and surrounding fibrocytes of the inner *Lamina propria* and the outer *Tunica adventitia*, and a highly distensible yet tightly sealing specialized inner epithelial lining. This urothelium features at the luminal side large binucleate superficial (S-) cells that exert barrier function at least partly due to expression of uroplakins (UPKs) that form crystalline plaques on the surface. Underneath are two layers of smaller intermediate (I-) and basal (B-) cells that serve as precursors in injury conditions and tether the underlying fibrocytes, respectively [1–4].

Although the tissue architecture of the ureter is much less complex compared to the adjacent kidney, our knowledge on the cellular and molecular programs that drive the growth and differentiation of this organ from a simple embryonic rudiment have only recently begun to be elucidated [5]. Cell lineage and marker analyses in the mouse have shown that the different epithelial and mesenchymal cell types of the ureter arise in a highly coordinated fashion from uncommitted progenitors that are established around embryonic day (E)11.5 from two independent precursor pools in the early metanephric field, the distal portion of the ureteric bud and its surrounding mesenchyme [4]. At E12.5, the initially homogenous ureteric mesenchyme is radially subdivided into an inner layer of large cuboidal cells, and an outer layer of tangentially oriented loosely organized cells. While the latter start to differentiate into adventitial fibrocytes from E13.5 onwards, the first maintain a bipotential character until E15.5 when they differentiate into SMCs, and subepithelial fibrocytes of the *Lamina propria*. Urothelial differentiation starts around E14.5 with the establishment of a common progenitor for S- and B-cells, the I-cell that expresses ΔNP63 and low levels of UPKs. At E15.5, first luminal cells downregulate ΔNP63 and express high levels of UPKs to become S-cells. KRT5^+^ B-cells are first recognized at E16.5. They substantially expand thereafter to constitute the major cell type of the adult urothelium (see S1 Fig for a scheme of the cellular composition of the embryonic and adult ureter) [4].

Survival, growth and differentiation of the ureteric mesenchyme and epithelium are tightly coupled and rely on the exchange of signals between and within the two tissues [5–7]. While our knowledge of the signaling systems that underlie urothelial development has remained scarce, WNTs, BMP4 and SHH have been identified as crucial signals for SMC differentiation in the mesenchyme [8–11]. SHH is expressed in the ureteric epithelium throughout development and is thought to act in a paracrine fashion onto the adjacent mesenchyme. Global or tissue-specific deletion of *Shh* from the epithelium resulted in reduced mesenchymal proliferation and delayed SMC differentiation, and culminated in hydroureter, i.e. dilatation of the ureter by urinary pressure [8, 11]. The molecular targets of SHH signaling are poorly understood. So far, expression of the genes encoding the transcription factor TSHZ3 and the signaling molecule BMP4 have been described to depend on SHH pathway activity in the ureteric mesenchyme [8, 12]. Both genes are essential for SMC differentiation arguing that they mediate some part of SHH function [9, 12]. Canonical WNT signaling is also required for SMC differentiation and may act in parallel to SHH in this process [10].

Here, we further explore the cellular and molecular functions of (S)HH signaling in the ureteric mesenchyme. We show that HH signaling is not only required for mesenchymal proliferation and differentiation but also prevents apoptosis in adventitial precursors and is essential for growth and differentiation of the ureteric epithelium. We provide evidence for a mesenchymal FOXF1-BMP4 module acting downstream of SHH in the execution of the proliferation and differentiation functions.

## Results

### Conditional inactivation of *Smo* in the ureteric mesenchyme results in hydroureter formation due to functional insufficiencies of its tissue compartments

To investigate the functional requirement of HH signaling in ureter development, we employed a conditional gene inactivation approach using a *Tbx18*^*cre*^ line [13] and a floxed allele of *Smo* (*Smo*^*fl*^) [14] which encodes a unique signal transducer of this pathway [15]. As previously reported, *Tbx18*^*cre*^ mediates recombination in the undifferentiated ureteric mesenchyme from E10.5 onwards, i.e. in the precursors of all differentiated cell types of the ureteric wall [10, 16].

**Fig 1:**
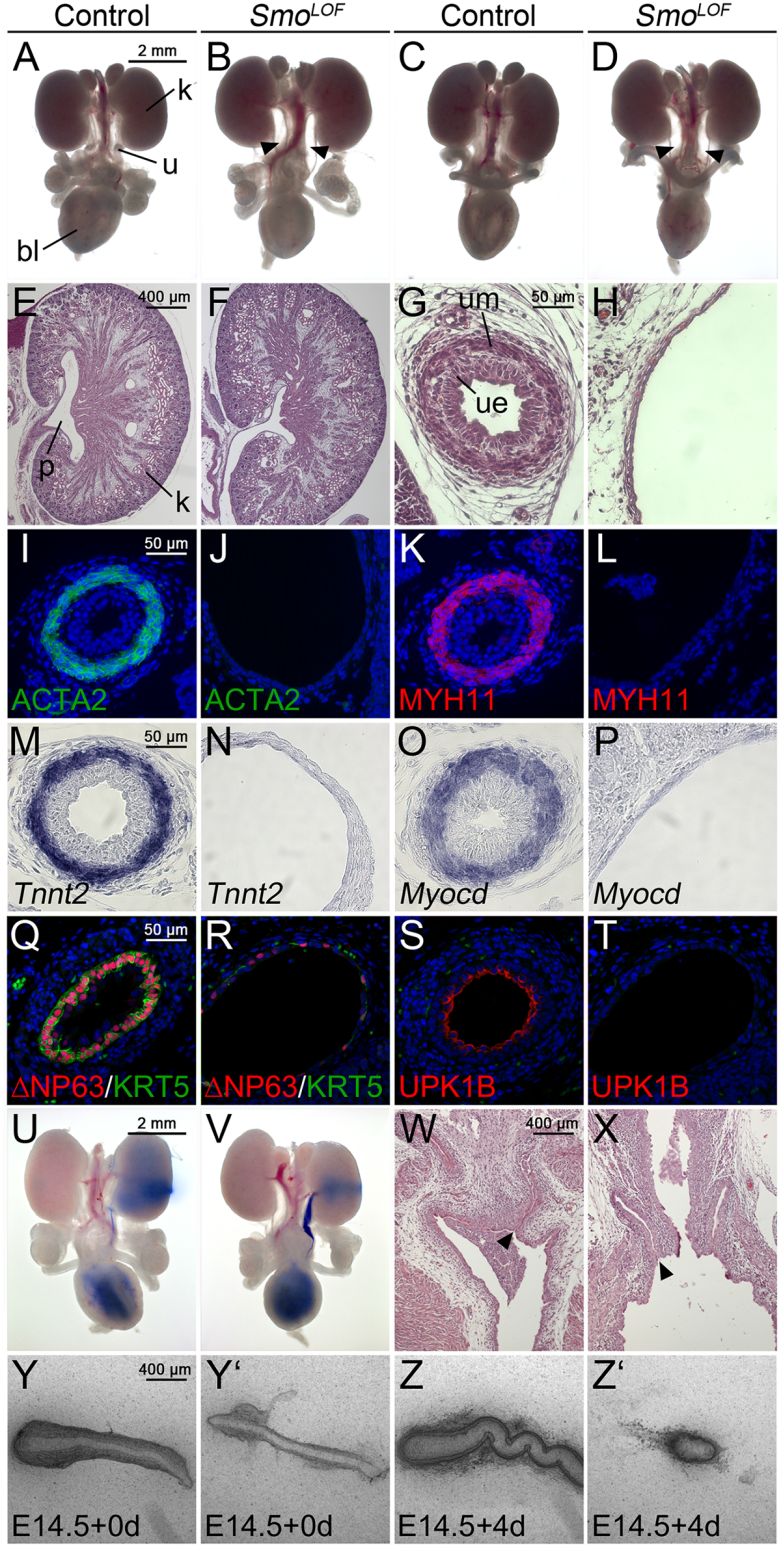
*Tbx18*^*cre/+*^;*Smo*^*fl/fl*^ (*Smo*^*LOF*^) embryos exhibit severe ureter defects at E18.5. (A-D) Morphology of whole urogenital systems of male (A,B) and female (C,D) embryos. Arrows point to hydroureters in *Smo*^*LOF*^ embryos. (E-H) Hematoxylin and Eosin (HE) stainings on midsagittal sections of the kidney (E,F) and of the proximal ureter (G,H). (I-T) Cytodifferentiation of the ureteric mesenchyme (I-P) and urothelium (Q-T) is compromised in *Smo*^*LOF*^ embryos as shown by immunofluorescence (I-L,Q-T) or RNA *in situ* hybridization (M-P) analysis on transverse sections of the proximal ureter. (U-X) Absence of physical obstruction in the *Smo*^*LOF*^ ureter as revealed by ink injection experiments (U,V) and HE stainings of the vesicoureteral junction (W,X). (Y-Z’) Explants of E14.5 ureters after 0 and 4 days of culture. Genotypes and markers are as shown.

At E18.5, urogenital systems of *Tbx18*^*cre/+*^;*Smo*^*fl/fl*^ (*Smo*^*LOF*^) mice displayed complete bilateral hydroureter with full penetrance in both sexes (Fig 1A–1D, S1 Table for a list of numbers, genotypes and conditions used for this and the other experiments). Histological analyses revealed a reduced pelvic space in mutant kidneys. The ureter was strongly dilated and featured a mono-layered urothelium that was surrounded by fibroelastic material (Fig 1E–1H). Expression of the structural components of the SMC layer ACTA2, MYH11 and *Tnnt2* as well as of the key regulator of the SMC transcriptional program *Myocd* was completely absent in the mutant ureter (Fig 1I–1P). Differentiation of urothelial cell types was also severely compromised in the mutant ureter as indicated by a strong decrease in expression of the B- and I-cell markers KRT5 and ΔNP63, and absence of superficial UPK1B expression (Fig 1Q–1T). To test for the contiguity of the ureteric lumen and the patency of the uretero-pelvic and vesicular junctions, we injected ink into the renal pelvis. Under conditions of increased hydrostatic pressure, the ink readily drained to the bladder both in control and *Smo*^*LOF*^ urogenital systems. Furthermore, the ureters in *Smo*^*LOF*^ embryos terminated in the bladder neck as in the control, arguing together that physical obstruction does not cause or contribute to the hydroureter phenotype in *Smo*^*LOF*^ urogenital systems (Fig 1U–1X). Control ureters explanted at E14.5 and cultured for 4 days elongated and performed unidirectional peristaltic contractions within 2 days of culture. In contrast, *Smo*^*LOF*^ ureters were strongly hypoplastic when explanted and degenerated in culture without showing any signs of contractile activity (Fig 1Y and 1Z’). Hence, lack of *Smo*, i.e. of HH signaling, in the ureteric mesenchyme results in tissue hypoplasia, a complete lack of differentiated mesenchymal and epithelial cell types and hydroureter formation at birth.

### *Smo*^*LOF*^ ureters show early hypoplasia and do not initiate the SMC and urothelial differentiation programs

**Fig 2:**
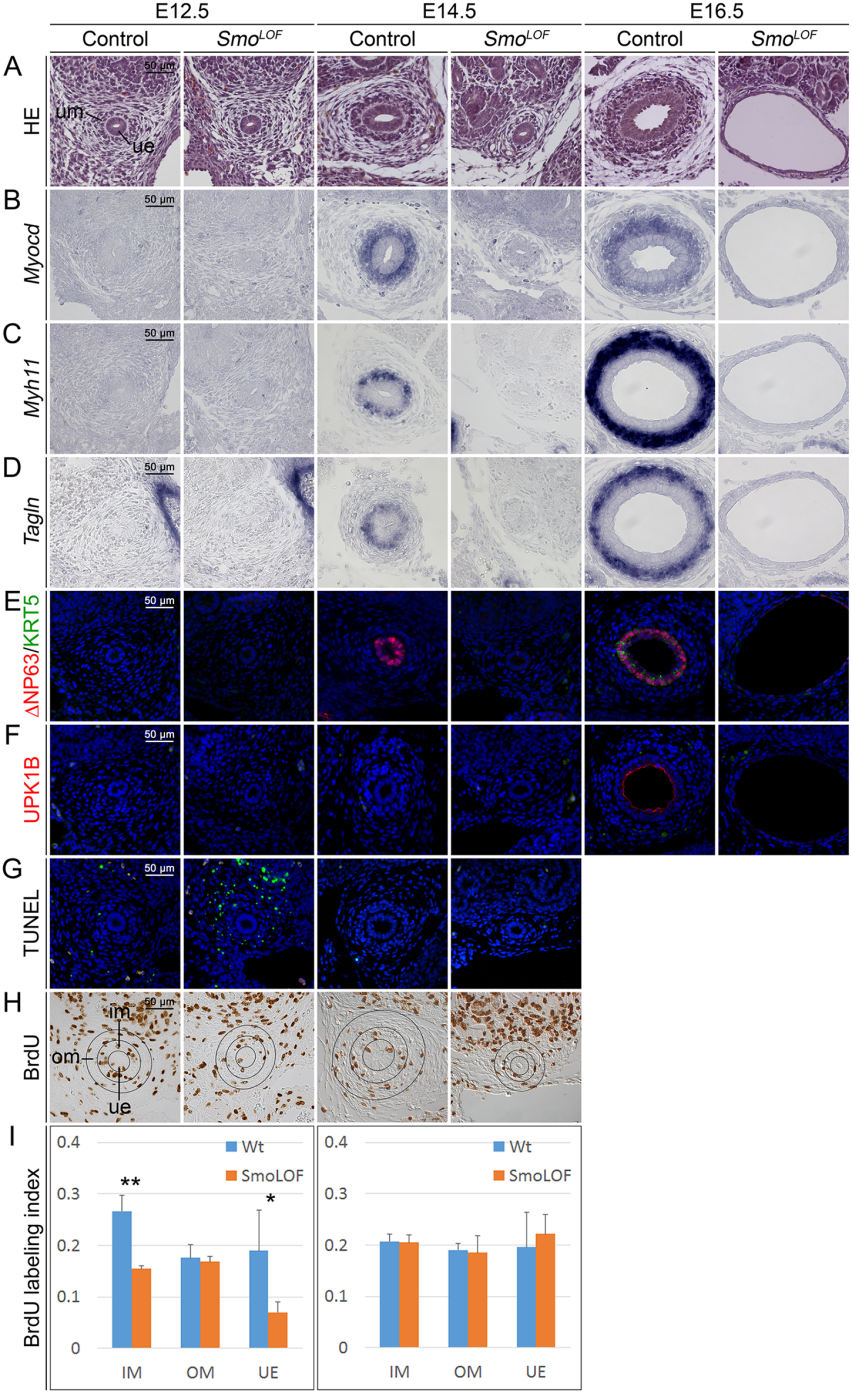
Ureter anomalies arise early in *Tbx18*^*cre/+*^;*Smo*^*fl/fl*^ (*Smo*^*LOF*^) embryos. (A) Hematoxylin and Eosin (HE) stainings of transverse sections of the proximal ureter at E12.5, E14.5, and E16.5. (B-D) Cytodifferentiation of the ureteric mesenchyme into SMCs as detected by *in situ* hybridization of expression of SMC marker genes, and (E,F) cytodifferentiation of the ureteric epithelium as detected by immunofluorescence of B-, I- and S-cell markers fail in *Smo*^*LOF*^ embryos. Nuclei are counterstained with DAPI (blue). (G) Cell death as detected by the TUNEL assay (green) occurs in the outer mesenchymal region of *Smo*^*LOF*^ ureters at E12.5. Nuclei are counterstained with DAPI. (H) Determination of cellular proliferation by the BrdU incorporation assay on transverse sections of the proximal ureter at E12.5 and E14.5. Black circles in H mark the epithelium (ue) and the inner (im) and outer (om) mesenchymal compartments of the ureter that were analyzed to quantify proliferation. Proliferation in *Smo*^*LOF*^ embryos is reduced in the inner mesenchymal region and the epithelium of the E12.5 ureter. (I) Quantification of BrdU-positive cells. E12.5 (n = 3), wildtype versus mutant: IM, 0.267±0.030 vs 0.155±0.006, P = 0.007; OM, 0.176±0.025 vs 0.169±0.009, P = 0.709; UE, 0.190±0.079 vs 0.071±0.019, P = 0.019. E14.5 (n = 4), wildtype versus mutant: IM, 0.208±0.015 vs 0.205±0.014, P = 0.839; OM, 0.191±0.013 vs 0.185±0.033, P = 0.774; UE, 0.196±0.068 vs 0.222 ±0.037, P = 0.526. Values are displayed as mean ± sd. *, P≤0.05; **, P≤0.01; two-tailed Student’s t-test.

To characterize the onset and progression of the growth and differentiation defects in *Smo*^*LOF*^ ureters, we performed histological and molecular analysis at earlier embryonic stages (Fig 2A–2F). At E12.5, *Smo*^*LOF*^ ureters appeared histologically unremarkable. The mono-layered ureteric epithelium was surrounded by up to two layers of spherical, densely packed mesenchymal cells with a clear compartment boundary to the outer spindle-shaped and radially oriented cells as in the control, indicating that initial tissue patterning of the ureteric mesenchyme was normal (Fig 2A, left panel). At E14.5, the urothelium of control embryos started to stratify and appeared occasionally double-layered, the compartmentalization of the ureteric mesenchyme was enhanced. In contrast, the *Smo*^*LOF*^ urothelium remained mono-layered and the mesenchymal cell mass was sparse (Fig 2A, middle panel). At E16.5, the onset of substantial urine production in the embryonic kidney, *Smo*^*LOF*^ mutants showed a strongly dilated ureter with a mono-layered urothelium surrounded by loosely packed fibrocytes (Fig 2A, right panel).

To characterize the initiation and progression of ureteric SMC differentiation, we analyzed the expression of the regulatory gene *Myocd* as well as of the SMC structural genes *Myh11* and *Tagln*. *Myocd* expression was homogenously strong in the wildtype at E14.5 and E16.5 whereas *Myh11* and *Tagln* expression was spotty at E14.5 and became stronger at E16.5. *Smo*^*LOF*^ ureters never expressed any of these markers, indicating that SMC differentiation was not initiated in the mutants (Fig 2B–2D). Urothelial differentiation started in the wildtype at E14.5 with the expression of ΔNP63 and continued at E16.5 with the appearance of ΔNP63^+^KRT5^+^ B-cells and UPK1B^+^ΔNP63^-^ S-cells. In *Smo*^*LOF*^ mutant ureters, only few cells expressed ΔNP63 at low levels at E16.5, KRT5 expression and UPK1B was not observed (Fig 2E and 2F). Taken together, *Smo*^*LOF*^ ureters develop severe mesenchymal and epithelial hypoplasia and fail to initiate the SMC and urothelial differentiation programs.

### HH signaling is required for survival and proliferation programs in the undifferentiated ureteric tissues

To address the cellular causes for the severe tissue hypoplasia observed in *Smo*^*LOF*^ ureters, we examined survival and proliferation in the epithelial and mesenchymal tissue compartments (Fig 2G–2I). We used the terminal dUTP nick end-labeling (TUNEL) assay to detect apoptotic bodies. At E12.5, only few apoptotic bodies were detected in control specimens whereas *Smo*^*LOF*^ ureters showed numerous strong signals specifically in the outer mesenchymal compartment. At E14.5, no signals were detected in either control or mutant ureters (Fig 2G). To assess proliferation rates, we performed bromodeoxyuridine (BrdU) incorporation assays. At E12.5, *Smo*^*LOF*^ ureters showed significantly decreased proliferation in the inner mesenchymal compartment and the epithelium; outer mesenchymal cells proliferated at normal rates. At E14.5, no changes in proliferation were observed in *Smo*^*LOF*^ ureters (Fig 2H and 2I). We conclude that *Smo* is required for the survival of cells of the outer mesenchymal compartment and the proliferation of inner mesenchymal and epithelial cells, specifically at E12.5.

### *Smo*^*GOF*^ ureters develop severe mesenchymal hyperplasia but show normal tissue patterning and differentiation

**Fig 3:**
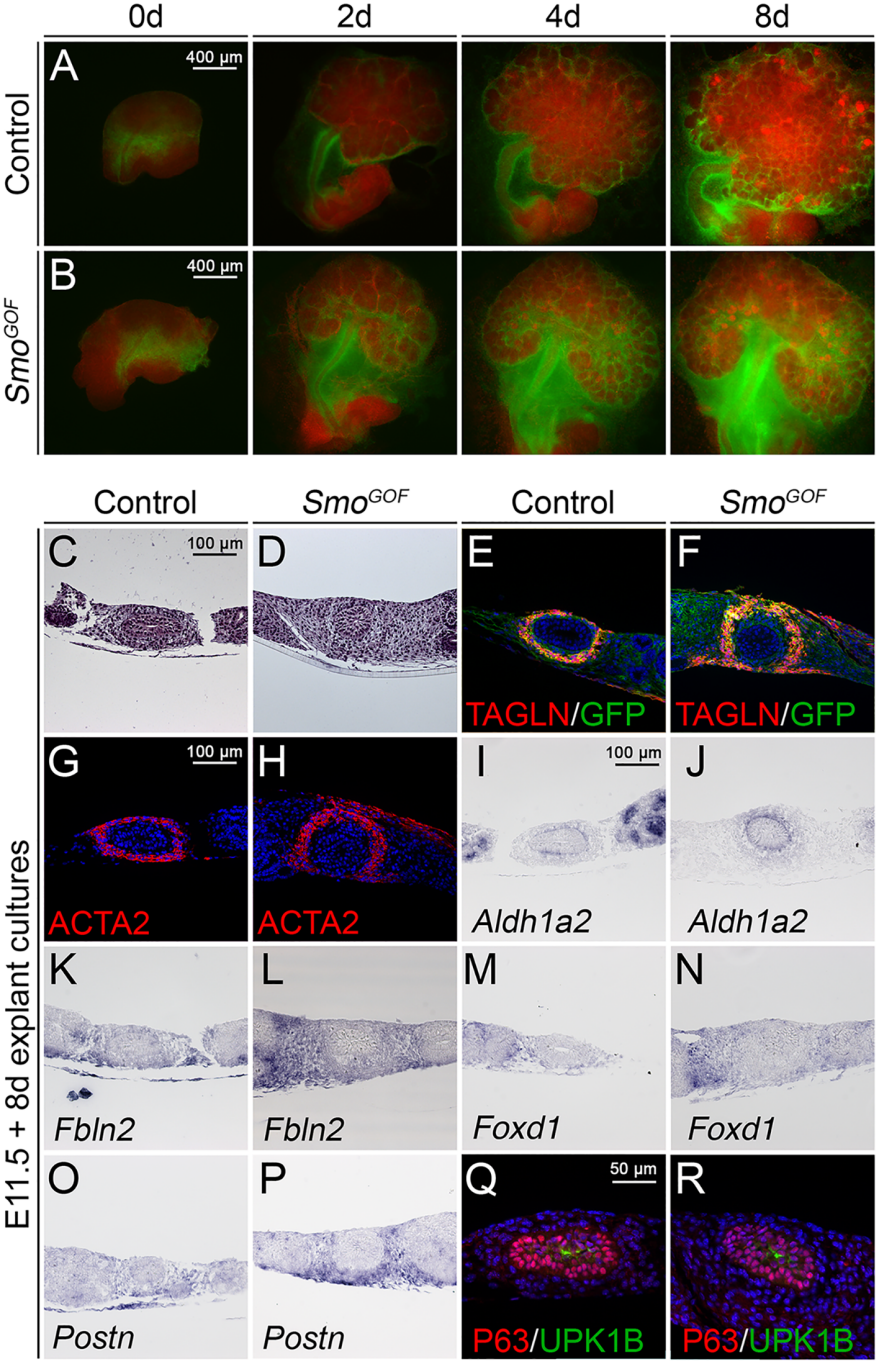
Conditional activation of *Smo* in the ureteric mesenchyme leads to mesenchymal hyperplasia but normal tissue patterning and differentiation. (A,B) GFP/RFP epifluorescence in E11.5 kidney/ureter explants of *Tbx18*^*cre/+*^;*R26*^*mTmG/+*^ (control) and *Tbx18*^*cre/+*^;*R26*^*mTmG/SmoM2*^ (*Smo*^*GOF*^) embryos after 0, 2, 4 and 8 d of culture. (C-R) Analysis of proximal sections of ureter explants of E11.5 wildtype and *Tbx18*^*cre/+*^;*R26*^*mTmG/SmoM2*^ embryos cultured for 8 d by Hematoxylin and Eosin staining (C,D), immunofluorescence of SMC markers TAGLN and ACTA2 (E-H) and urothelial markers ΔNP63/UPK1B (Q,R), and *in situ* hybridization analysis of the lamina propria marker *Aldh1a2* (I,J) and the adventitial fibroblast markers *Fbln2*, *Foxd1* and *Postn* (K-P).

The analysis of the *Smo* loss-of-function phenotype revealed a critical requirement of HH signaling in survival, growth and differentiation of the ureter. To test for a possible sufficiency in these cellular processes we performed a complementary gain-of-function study by conditional (*Tbx18*^*cre*^-mediated) misexpression of a constitutive active form of SMO from the *Rosa26* locus (*R26*^*SmoM2*^) [17] in the ureteric mesenchyme. *Tbx18*^*cre/+*^;*R26*^*SmoM2/+*^ (*Smo*^*GOF*^) embryos died around E12.5 due to cardiovascular defects [18]. To circumvent this lethality and enable an endpoint analysis of ureter differentiation, we explanted E11.5 kidney rudiments and cultured them for 8 days. We took advantage of a membrane-bound GFP reporter from the *Rosa26*^*mTmG*^ reporter line [19] to visualize the descendants of the undifferentiated ureteric mesenchyme after *Tbx18*^*cre*^-mediated recombination. In *Tbx18*^*cre/+*^;*R26*^*mTmG/+*^ control explants, GFP^+^ cells initially localized to a band of mesenchymal cells that surrounded the ureter stalk and separated the metanephric mesenchyme from the nephric duct. In the following days, GFP^+^ cells became restricted to a condensed cell layer directly adjacent to the ureteric epithelium and to stromal cells of the medial kidney cortex as previously reported [16]. In *Smo*^*GOF*^ (*Tbx18*^*cre/+*^;*R26*^*mTmG/SmoM2*^) explants, GFP^+^ cells localized to these domains as well but additionally persisted in the lateral ureteric mesenchymal region to form a large ectopic cell mass after 4 and 8 days of culture (Fig 3A and 3B). Histological analysis on proximal ureter sections of *Smo*^*GOF*^ E11.5 + 8d explants confirmed severe mesenchymal hyperplasia (Fig 3C and 3D). Coimmunofluorescence analysis of GFP and the SMC markers TAGLN and ACTA2 revealed correct mesenchymal patterning into a GFP^+^TAGLN^-^ACTA2^-^ cell layer adjacent to the urothelium, a medial GFP^+^TAGLN^+^ACTA2^+^ SMC layer and an outer largely expanded coat of GFP^+^TAGLN^-^ACTA2^-^ cells in *Smo*^*GOF*^ explants (Fig 3E–3H). Expression of *Aldh1a2* identified the inner mesenchymal layer as the *Lamina propria* [10]. The strongly expanded outer mesenchymal cell layer expressed *Fbln2*, *Foxd1* and *Postn*, markers for adventitial fibrocytes in the wildtype (Fig 3I–3P) [4]. Urothelial differentiation as analyzed by ΔNP63 and UPK1B expression was unaltered (Fig 3Q and 3R). We conclude that mis- and overactivation of HH signaling in the ureteric mesenchyme leads to massive hyperplasia of the adventitial cell layer but does not affect tissue patterning or cell differentiation programs.

### HH signaling is sufficient to induce survival and proliferation of the ureteric mesenchyme

**Fig 4:**
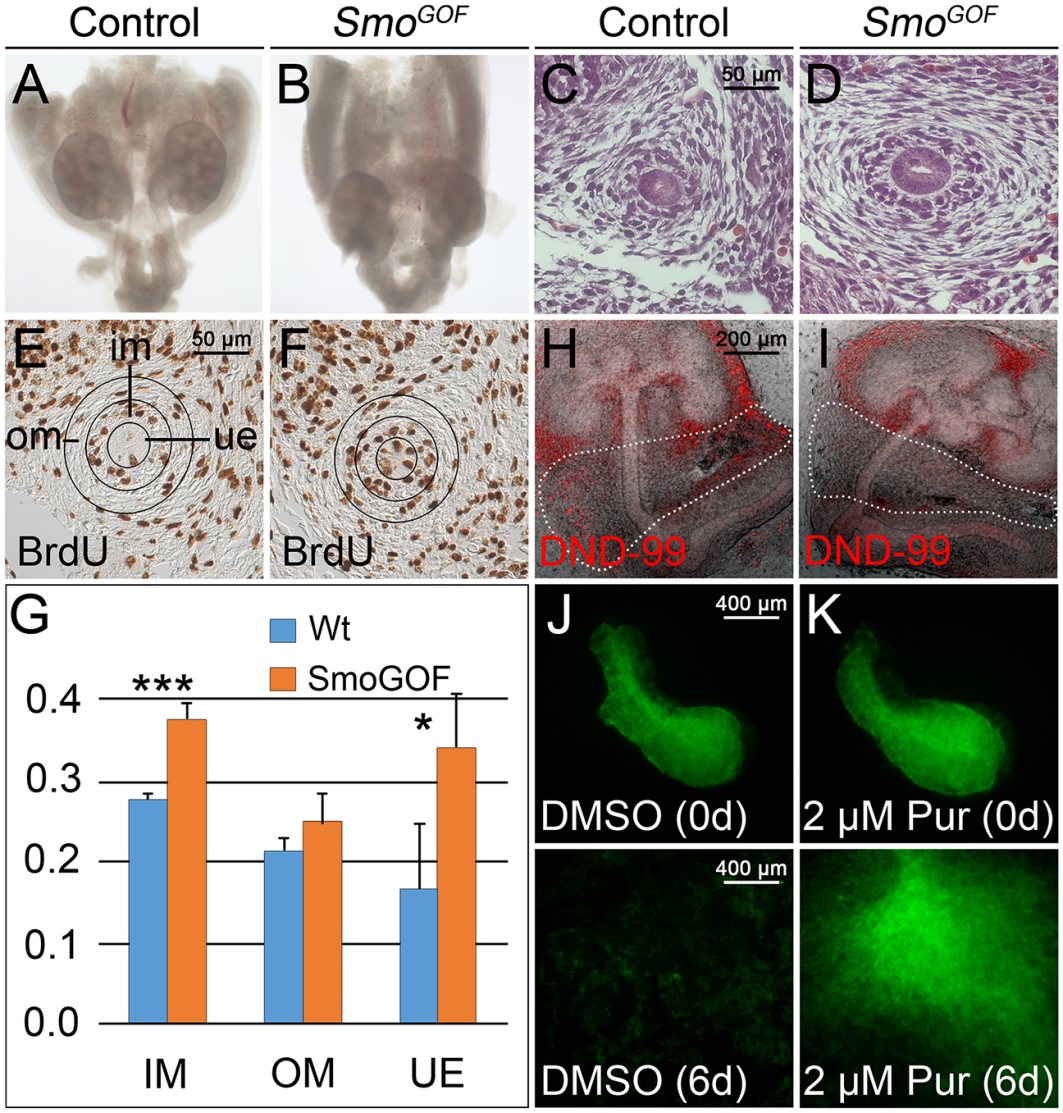
HH signaling is sufficient to maintain survival and proliferation of the ureteric mesenchyme. (A,B) Morphology of whole urogenital systems of wildtype (control) and *Tbx18*^*cre/+*^;*R26*^*mTmG/SmoM2*^ (*Smo*^*GOF*^) embryos at E12.5. (C,D) Hematoxylin and Eosin staining on transverse sections of the proximal ureter at E12.5. (E,F) Determination of cellular proliferation by the BrdU incorporation assay on transverse sections of the proximal ureter at E12.5. Black circles in E and F mark the epithelium (ue) and the inner (im) and outer (om) mesenchymal compartments of the ureter that were analyzed to quantify proliferation. (G) Quantification of BrdU-positive cells. E12.5 (n = 4), wildtype versus mutant: IM, 0.276±0.006 vs 0.375±0.020, P = 0.0001; OM, 0.214±0.015 vs 0.250±0.034, P = 0.0966; UE, 0.167±0.080 vs 0.341±0.066, P = 0.0211. Values are displayed as mean ± sd. *, P≤0.05; ***, P≤0.001 two-tailed Student’s t-test. (H,I) Analysis of cell death by Lysotracker (DND-99) incorporation of E11.5 control and *Smo*^*GOF*^ explants after 1 day of culture. (J,K) GFP epifluorescence of E12.5 *Tbx18*^*cre/+*^;*R26*^*mTmG/+*^ ureteric mesenchyme that was mechanically separated from the ureteric epithelium and cultured for 6 days with DMSO or 2 μM purmorphamine (Pur).

To unravel the cellular cause of tissue hyperplasia in *Smo*^*GOF*^ ureters, we analyzed histology, cell proliferation and apoptosis at the onset of ureter development. At E12.5, *Smo*^*GOF*^ ureters were shortened and surrounded by a large mass of fibrous tissue (Fig 4A and 4B). On the histological level, the subdivision of the ureteric mesenchyme into an inner region with large cuboidal cells and an outer region of more loosely organized tangentially oriented cell bodies was normal but the outer mesenchymal domain appeared strongly expanded (Fig 4C and 4D). The BrdU incorporation assay revealed significantly increased proliferation in the epithelium and the inner mesenchymal domain of *Smo*^*GOF*^ ureters at E12.5 (Fig 4E–4G). Incorporation of Lysotracker (DND-99), a fluorogenic marker for acidified organelles [20], into whole E11.5 explants after 1 day of culture indicated absence of apoptotic cells in the entire ureteric mesenchyme, with the lateral aspect of *Smo*^*GOF*^ explants being most prominently affected (Fig 4H and 4I). Moreover, mesenchyme mechanically separated from the ureteric epithelium and cultured for 6 days in the presence of 2 μM of the SMO agonist purmorphamine [21] survived while DMSO treated control explants died (Fig 4J and 4K). These experiments show that tissue hyperplasia after mis- and overactivation of HH signaling in the ureteric mesenchyme is caused by a combination of reduced cell death in adventitial precursors and increased cell proliferation in SMC progenitors and the epithelial compartment.

### *Foxf1* is a target of HH signaling in the ureteric mesenchyme

To get insight into the spectrum of genes controlled by HH signaling in the ureteric mesenchyme, we wished to determine the global transcriptional changes caused by inhibition of the pathway in the ureter. Rather than comparing mutant (*Smo*^*LOF*^) and wildtype ureters, we deemed that pharmacological inhibition of HH signaling by the SMO antagonist cyclopamine [22, 23] in ureter explant cultures would provide a better handle to identify primary transcriptional changes. Treatment of E12.5 ureters with 10 μM cyclopamine led to a robust down-regulation of expression of the direct target of HH signaling *Ptch1* in the ureteric mesenchyme [24] (S2 Fig). Moreover, administration of 10 μM cyclopamine to E11.5 kidney/ureter explants for 2 days led to a complete loss of ureteric SMC differentiation after 8 days of culture while inhibition in later time intervals left this differentiation program unaffected (S3 Fig). Since these findings delimited the requirement of HH signaling in the ureteric mesenchyme to E11.5 to E13.5, we decided to treat E12.5 ureters with 10 μM cyclopamine for 18 h to perform microarray profiling of differential gene expression with untreated controls.

**Fig 5:**
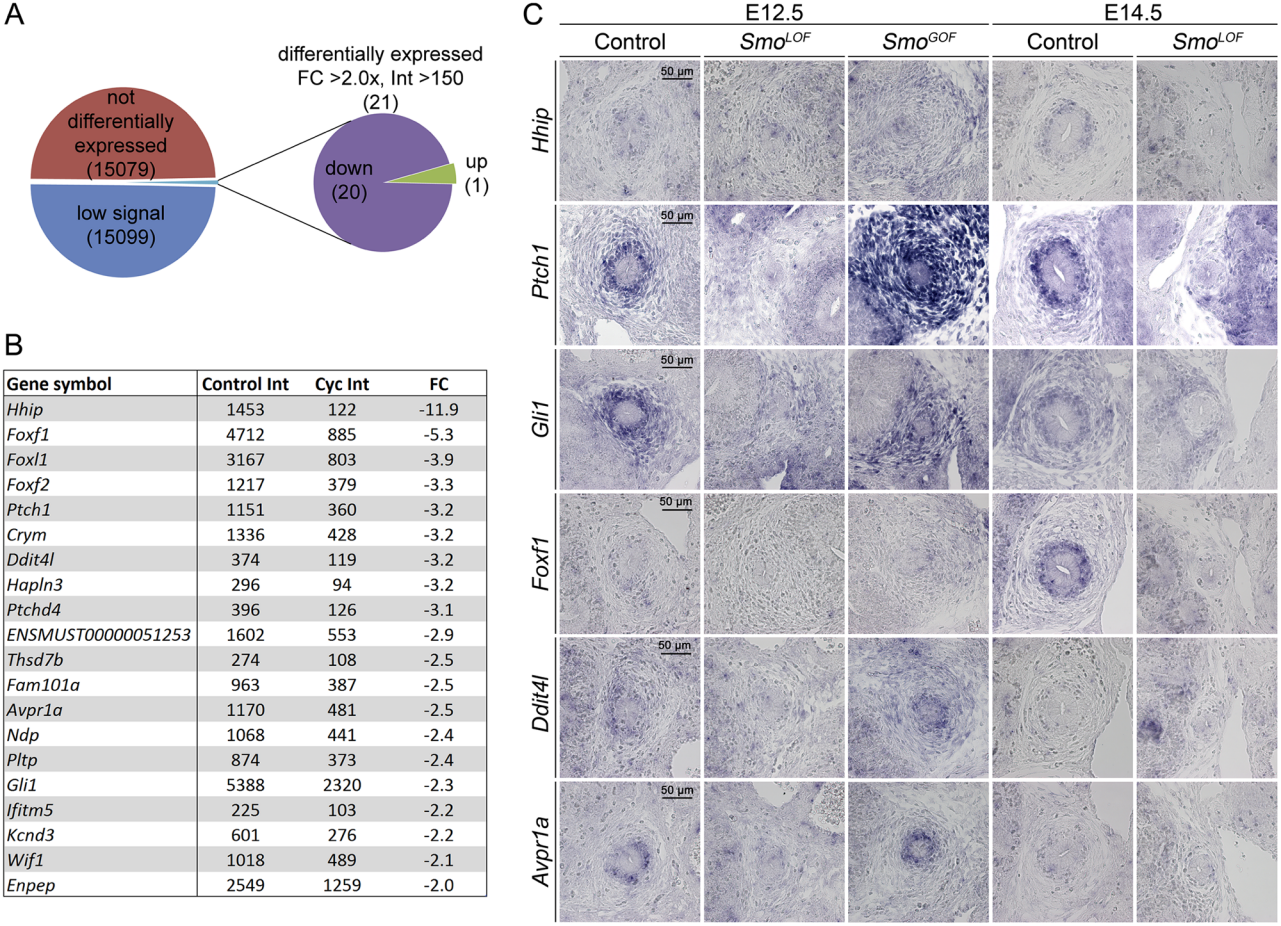
Microarray analysis detects transcriptional changes after loss of HH signaling in the ureteric mesenchyme. (A) Pie-chart summarizing the results from the microarray analysis of E12.5 ureters explanted and treated with DMSO or 10 μM cyclopamine for 18 h filtered with an intensity (Int) threshold of 150 and a fold change (FC) cut-off of 2.0. (B) Table of the downregulated transcripts. Shown are average intensities of transcripts in control and cyclopamine treated ureters and average fold changes (FC) of RNA intensities between the pools in two independent experiments. (C) *In situ* hybridization analysis of expression of microarray candidates on proximal ureter sections of control, *Tbx18*^*cre/+*^;*Smo*^*fl/fl*^ (*Smo*^*LOF*^) and *Tbx18*^*cre/+*^;*R26*^*mTmG/SmoM2*^ (*Smo*^*GOF*^) ureters at E12.5 and E14.5.

Using an intensity threshold of 150, we identified in two independent pools of treated and untreated ureters a small set of 20 genes that were consistently more than 2-fold downregulated and only one gene that was upregulated in expression in cyclopamine treated ureters (Fig 5A and S2 Table). Among the downregulated transcripts were the three *bona fide* SHH target genes *Hhip* (-11.9x), *Ptch1* (-3.2x) and *Gli1* (-2.3x), confirming the specificity of our assay [24–28]. Another group of prominently downregulated transcripts comprised three members of the Forkhead transcription factor family, *Foxf1* (-5.3x), *Foxl1* (-3.9x) and *Foxf2* (-3.3x), which have been reported to be primary SHH target genes in several other contexts [29–31] (Fig 5B).

**Fig 6:**
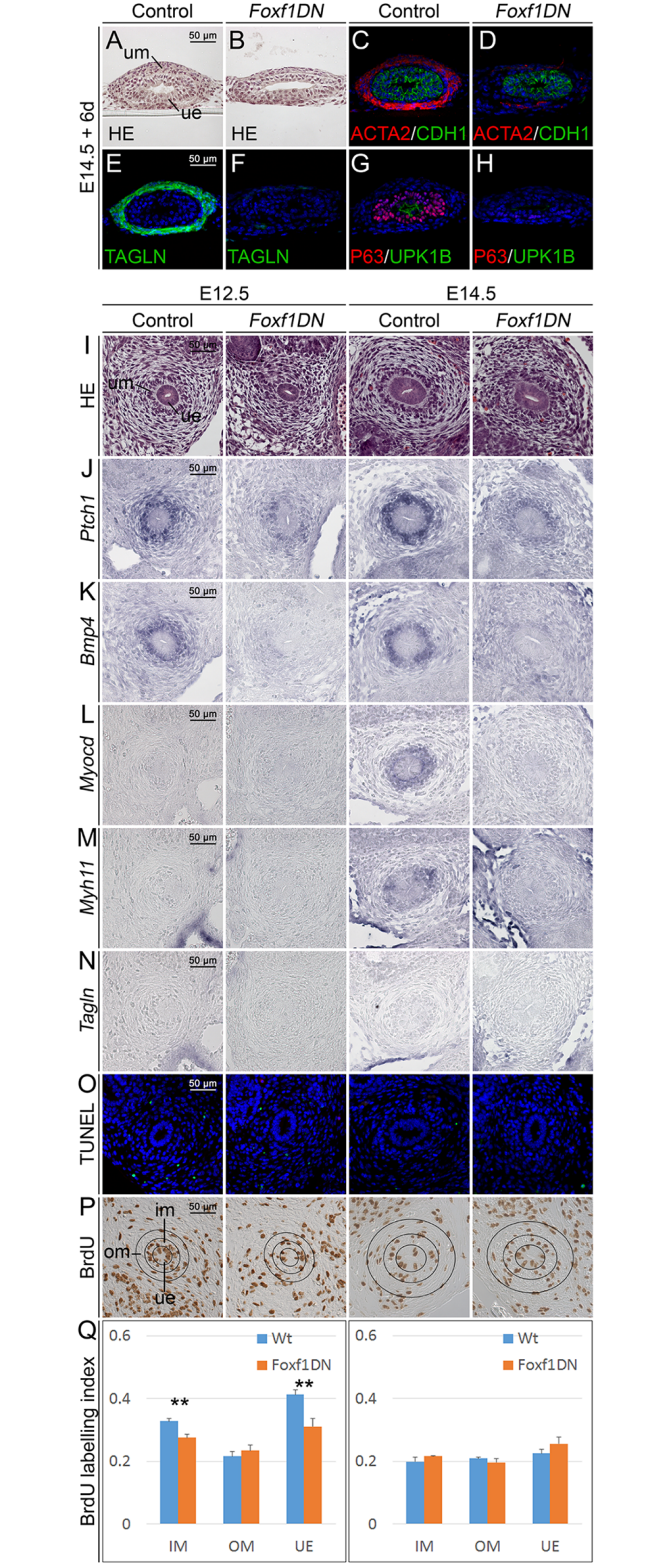
*Foxf1* is a crucial factor for ureter development. (A-H) Histological and molecular analysis of E14.5 control and *Tbx18*^*cre/+*^;*Hprt*^*Foxf1DN/y*^ (*Foxf1DN*) ureters after 6 d of culture. (A,B) Hematoxylin and Eosin (HE) stainings and (C-H) analysis of cytodifferentiation by immunofluorescence for ACTA2/CDH1 (C,D), TAGLN (E,F) and ΔNP63/UPK1B (G,H) on proximal ureter sections. (I-Q) Histological, cellular and molecular analysis of control and *Tbx18*^*cre/+*^;*Hprt*^*Foxf1DN/y*^ (*Foxf1DN*) ureters at E12.5 and E14.5. (I) Hematoxylin and Eosin staining on transverse sections of the proximal ureter at E12.5 and E14.5. (J-N) RNA *in situ* hybridization analysis for *Ptch1* (J), *Bmp4* (K), *Myocd* (L), *Myh11* (M) and *Tagln* (N) expression on transverse sections of the proximal ureter at E12.5 and E14.5. (O-Q) Analysis of cell death by the TUNEL assay (O), and of cellular proliferation by the BrdU incorporation assay (P) on transverse sections of the proximal ureter at E12.5 and E14.5. Black circles in H mark the epithelium (UE) and the inner (IM) and outer (OM) mesenchymal compartments of the ureter for which proliferation was quantified. (Q) Quantification of BrdU-positive cells. E12.5 (n = 3), wildtype versus mutant: IM, 0.329±0.007 vs 0.276±0.011, P = 0.002; OM, 0.216±0.015 vs 0.234±0.019, P = 0.310; UE, 0.412±0.016 vs 0.311±0.026, P = 0.004. E14.5 (n = 3), wildtype versus mutant: IM, 0.199±0.013 vs 0.216±0.003, P = 0.153; OM, 0.209±0.005 vs 0.197±0.013, P = 0.288; UE, 0.225±0.014 vs 0.256±0.020, P = 0.144. Values are displayed as mean ± sd. **, P≤0.01; two-tailed Student’s t-test.

To validate our microarray results and determine the spatial expression of selected candidates we performed *in situ* hybridization analysis on proximal sections of E12.5 control *Smo*^*LOF*^ and *Smo*^*GOF*^ and of E14.5 control and *Smo*^*LOF*^ ureters (Fig 5C and S4 Fig). *Hhip* expression was barely detectable at E12.5 and E14.5 in the ureteric mesenchyme of wildtype embryos but was abrogated in *Smo*^*LOF*^ and weakly induced in *Smo*^*GOF*^ ureters. Expression of *Ptch1* and *Gli1* was strong in the inner layer of the ureteric mesenchyme at E12.5 in the control. Expression was completely lost in *Smo*^*LOF*^ ureters, but induced in the entire ureteric mesenchyme in *Smo*^*GOF*^ ureters at this stage. At E14.5, the expression of *Ptch1* and *Gli1* in the inner mesenchymal compartment was HH signaling-dependent, whereas outer mesenchymal cells maintained normal levels of *Gli1*. *Foxf1* expression was detectable at E14.5 in the inner mesenchymal compartment of wildtype embryos. Expression was completely lost in *Smo*^*LOF*^ ureters at this stage but was not induced in *Smo*^*GOF*^ ureters at E12.5. Expression of *Ddit4l* (-3.2x) and *Avpr1a* (-2.5x) was detected in the inner mesenchymal region of controls at E12.5, and was down-regulated in *Smo*^*LOF*^ but not induced in *Smo*^*GOF*^ ureters (Fig 6C). The sensitivity of the method was not sufficient to detect expression of *Foxl1*, *Foxf2*, *Crym*, *Ndp* and *Wif1* (S4 Fig).

Previous work suggested that *Bmp4* and *Tshz3* are targets of HH signaling [8, 12], and that *Bmp4*, *Tshz3*, *Tcf21*, *Tbx18*, *Sox9* and canonical WNT signaling are functionally involved in SMC differentiation in the ureter or in other contexts [9, 12, 13, 32, 33]. In our microarray, expression of *Bmp4* and *Tcf21* was 1.5 fold down-regulated, expression of *Tshz3*, *Tbx18* and *Sox9* was unchanged, and canonical WNT signaling as seen by expression of the *bona fide* target gene *Axin2* {Jho, 2002 #36} was slightly upregulated (S5A Fig). *In situ* hybridization analysis showed that expression of *Bmp4* and *Tcf21* in the ureteric mesenchyme was strongly reduced in *Smo*^*LOF*^ ureters at E12.5 and E14.5. Interestingly, *Tcf21* expression was expanded into the entire ureteric mesenchyme in *Smo*^*GOF*^ ureters whereas *Bmp4* expression remained confined to the entire mesenchymal region albeit at increased levels. *Tshz3*, *Tbx18* and *Sox9* exhibited slightly reduced expression in the ureteric mesenchyme in *Smo*^*LOF*^ ureters (possibly due to tissue hypoplasia), and were weakly expanded to the outer mesenchymal domain in *Smo*^*GOF*^ ureters. In E14.5 *Smo*^*LOF*^ ureters, their expression was maintained at low levels in the inner mesenchymal region. Expression of *Axin2* in the inner mesenchymal cell layer appeared unaffected by loss or gain of HH signaling in the ureteric mesenchyme (S5B Fig). Together, these assays identify *Foxf1*, *Ddit4l* and *Avpr1a* as novel targets of HH signaling in the ureteric mesenchyme. Expression of *Bmp4* and *Tcf21* may indirectly depend on HH signaling whereas *Tshz3*, *Tbx18*, *Sox9* and canonical WNT signaling are independent from this pathway.

### *Foxf1* is required for proliferation and cell differentiation in the developing ureter

Previous work identified prominent functions for *Foxf1* and *Foxf2* as targets of epithelial SHH signals in SMC differentiation of the intestinal mesenchyme [30]. Given that *Foxf1* is expressed in the ureteric mesenchyme at E14.5, i.e. prior to the onset of SMC and S-cell differentiation, we wondered whether this transcription factor mediates part of the function of HH signaling in these programs in the ureteric mesenchyme. To test this hypothesis, we used a conditional Cre/*loxP*-based transgenic approach to misexpress a dominant negative version of FOXF1 in the ureteric mesenchyme *in vivo*. We deemed such a strategy more efficient than a conditional knockout considering the possible redundancy of *Foxf1* and *Foxf2*. We generated the dominant negative version of FOXF1 by fusing the open reading frame of this transcriptional activator to a cDNA fragment harboring the strong transcriptional repression domain of the *Drosophila* ENGRAILED (ENG) protein [34, 35]. This coding region was followed by a fragment harboring an IRES-GFP sequence to allow visualization of misexpressing cells. The bicistronic transgene-cassette was integrated in the ubiquitously expressed X-chromosomal Hypoxanthine guanine phosphoribosyl transferase (*Hprt*) locus (*Hprt*^*Foxf1DN*^) [36, 37]. Transgene expression was driven by the *Tbx18*^*cre*^ line. Due to random X-chromosome inactivation, female *Tbx18*^*cre/+*^;*Hprt*^*Foxf1DN/+*^ embryos possessed a mosaic expression. Male *Tbx18*^*cre/+*^;*Hprt*^*Foxf1DN/y*^ (*Foxf1DN*) embryos expressed the transgene in a uniform manner and were subsequently used for phenotypic analysis.

Since *Foxf1DN* embryos died shortly after E14.5, we explanted E14.5 control and *Foxf1DN* ureters and cultured them for 6 days to assess tissue integrity and terminal differentiation. Hematoxylin and eosin staining of sections demonstrated mesenchymal hypoplasia and a reduction of the urothelium from three to two cell layers in cultured *Foxf1DN* ureters (Fig 6A and 6B). Immunofluorescence analysis revealed a strong reduction of TAGLN and ACTA2 expression and absence of ΔNP63/UPK1B indicating severely compromised mesenchymal and epithelial cell differentiation in *Foxf1DN* ureters at this stage (Fig 6C–6H).

To characterize the onset of these phenotypical changes, we analyzed earlier embryonic stages of *Foxf1DN* ureters. Hematoxylin and eosin stainings at E12.5 and E14.5 revealed no dramatic effects on overall tissue size in the epithelial and mesenchymal compartments of the mutant ureter. However sub-division of the ureteric mesenchyme into inner cuboidal SMC precursors and outer spindle shaped adventitial fibrocytes appeared less clear (Fig 6I). Expression of the HH target gene *Ptch1* was strongly reduced in the inner mesenchymal region at E12.5 and E14.5 indicating a possible role of FOXF1 as a feed-back activator of SHH signaling (Fig 6J). Strikingly, *Foxf1* was required to maintain *Bmp4* expression at E12.5 and E14.5, suggesting that *Foxf1* mediates *Bmp4* expression downstream of HH signaling (Fig 6K). *Tshz3*, *Tcf21*, *Tbx18*, *Sox9* and *Axin2* were normally expressed in *Foxf1DN* mutants, (S6 Fig). Importantly, SMC differentiation as analyzed by *Myocd*, *Myh11* and *Tagln* expression was not initiated in *Foxf1DN* ureters at E14.5 (Fig 6L–6N). Apoptosis in the ureter was not changed at either stage while proliferation in the inner mesenchymal region and the epithelium was reduced at E12.5 in *Foxf1DN* embryos (Fig 6O–6Q). We conclude that FOXF1 acts upstream of *Bmp4*, and is required to mediate the proliferation and differentiation but not the survival function of HH signaling in ureter development.

### FOXF1 suffices to induce the ureteric differentiation program downstream of HH signaling

**Fig 7:**
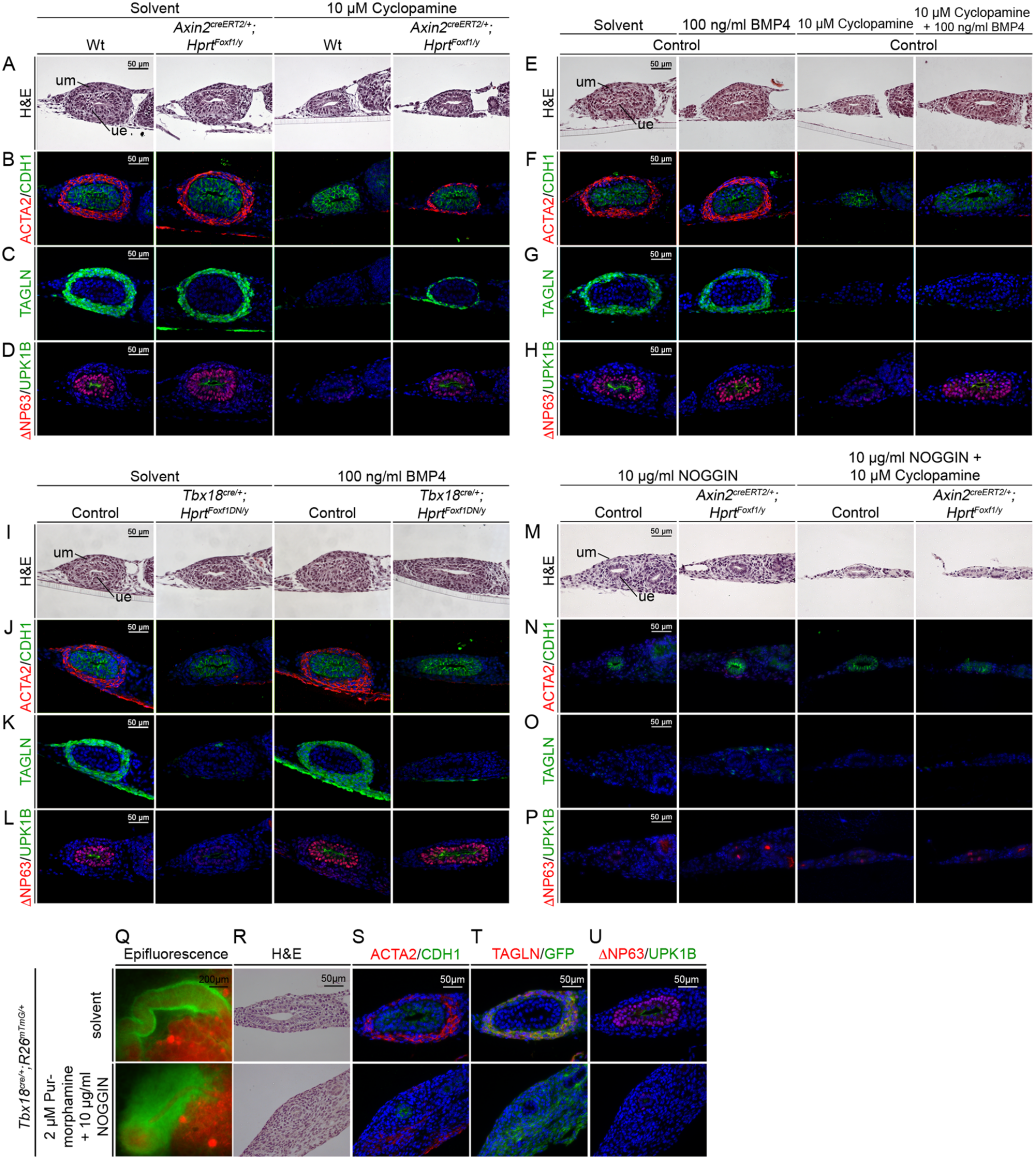
Analysis of the epistatic relationship of HH signaling, FOXF1 and BMP4 in cytodifferentiation of the ureter. (A-U) Ureters were explanted from E12.5 wildtype, *Axin2*^*creERT2/+*^;*Hprt*^*Foxf1/y*^, *Tbx18*^*cre/+*^;*Hprt*^*Foxf1DN/y*^ or *Tbx18*^*cre/+*^;*R26*^*mTmG/+*^ embryos and cultured for 6 d in the presence or absence of 10 μM cyclopamine, 100 ng/μl BMP4, 10 μg/ml NOGGIN, 2 μM Purmorphamine or solvent as indicated. Whole explants were documented by epifluorescence analysis (Q), or were sectioned and proximal regions analyzed by Haematoxylin and Eosin staining (A,E,I,M,R), by immunofluorescence (B-D,F-H,J-L,N-P,S-U) for the SMC marker ACTA2 together with the epithelial marker CDH1 (B,F,J,N,S), for the SMC marker TAGLN without (C,G,K,O) or with the lineage marker GFP (T), and for the urothelial markers ΔNP63/UPK1B (D,H,L,P,U).

To stringently test which of the various cellular functions of HH signaling in the ureter is mediated by FOXF1, we wished to restore *Foxf1* expression in ureter explants in which HH signaling was abolished by administration of cyclopamine. For this purpose, we employed the *Hprt* strategy again to generate an allele for conditional misexpression of *Foxf1* (*Hprt*^*Foxf1*^*)*. Since *Tbx18*^*cre/+*^;*Hprt*^*Foxf1/y*^ male embryos showed early embryonic lethality prior to the onset of kidney development at E10.5, we used an inducible *Axin2*^*creERT2*^ line that mediates recombination specifically in the inner mesenchymal domain of the ureter [4, 38]. *Axin2*^*creERT2/+*^;*Hprt*^*Foxf1/y*^ ureters explanted at E12.5 and cultured for 3 days in the presence of 500 nM 4-Hydroxy-Tamoxifen showed robust expression of *Foxf1* in the inner mesenchymal region and restored *Bmp4* and *Foxf1* expression after cyclopamine treatment proving the suitability of our approach (S7 Fig). E12.5 *Axin2*^*creERT2/+*^;*Hprt*^*Foxf1/y*^ ureters cultured for 6 days in the presence of 500 nM 4-Hydroxy-Tamoxifen with or without 10 μM cyclopamine were morphologically indistinguishable from wildtype controls indicating that reconstitution of *Foxf1* expression was not able to alleviate cyclopamine induced tissue hypoplasia in this setting (Fig 7A). However, SMC differentiation as analyzed by ACTA2 and TAGLN expression and urothelial differentiation as analyzed by ΔNP63 and UPK1B expression was rescued in cyclopamine treated *Axin2*^*creERT2/+*^;*Hprt*^*Foxf1/y*^ explants, indicating that FOXF1 controls these differentiation programs downstream of HH signaling (Fig 7B–7D).

To address the role of BMP4 as a possible mediator of the HH and FOXF1 function, we performed additional pharmacological manipulation experiments in ureter explant cultures. First, we cultured E12.5 ureters for 6 days with or without 100 ng/ml BMP4 and 10 μM cyclopamine. Interestingly, BMP4 was able to reduce cyclopamine-induced epithelial and mesenchymal hypoplasia and to completely restore epithelial differentiation. However, it was not sufficient to induce SMC differentiation (Fig 7E–7H). Similarly, BMP4 administration to E12.5 *Tbx18*^*cre*/+^;*Hprt*^*Foxf1DN/y*^ (*Foxf1DN)* explants did not rescue SMC differentiation but restored urothelial differentiation after 6 days of culture (Fig 7I–7L). To test the requirement of BMP4 downstream of HH signaling and FOXF1, we treated wildtype and *Axin2*^*creERT2*/+^;*Hprt*^*Foxf1/y*^ ureter explants with 10 μg/ml NOGGIN, which sequesters BMP4 from its receptor [39] or with a combination of NOGGIN and the SMO inhibitor cyclopamine. In all cases, expression of the SMC markers TAGLN and ACTA2, and of the urothelial markers ΔNP63 and UPK1B was completely abolished (Fig 7M–7P). Finally, when we co-treated wildtype ureters with the HH signaling activator puromorphamine and the BMP4 antagonist NOGGIN for 6 days, inner mesenchymal and epithelial cells were hypoplastic and did not differentiate. However, outer mesenchymal cells showed massive hyperplasia indicating that BMP4 is required downstream of HH signaling and FOXF1 for proliferation and differentiation of inner mesenchymal and epithelial cells but does not mediate the survival function in adventitial precursor cells.

## Discussion

**Fig 8:**
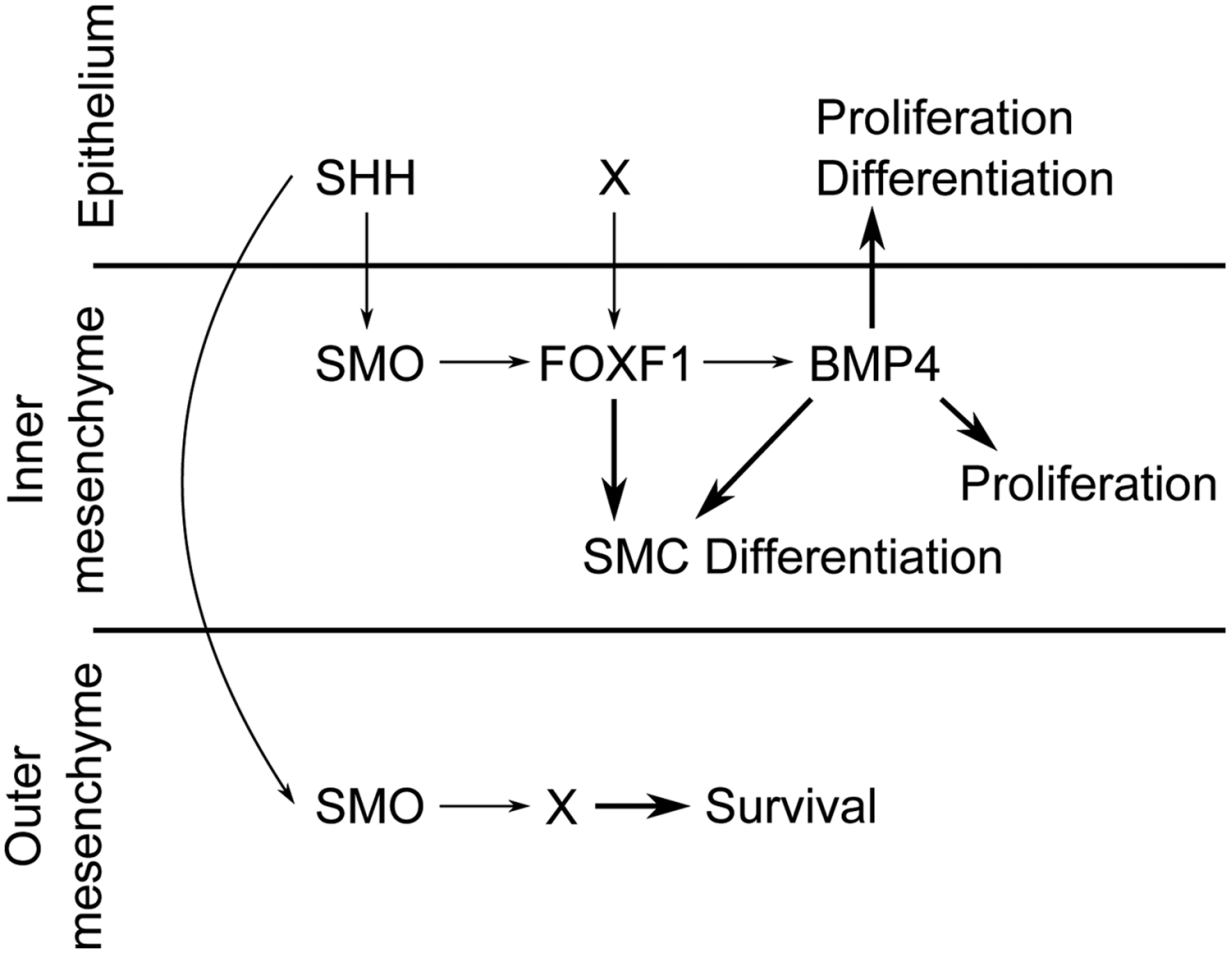
Model of how SHH and HH signaling direct various cellular programs in early ureter development. SHH is secreted from the ureteric epithelium and activates in the mesenchyme a SMO-dependent signaling pathway that induces expression of *Foxf1* in the inner mesenchymal domain. FOXF1, in turn activates and/or maintains expression of *Bmp4*. BMP4 regulates mesenchymal and epithelial proliferation, and epithelial differentiation. SMC differentiation depends both on *Bmp4* and *Foxf1*. Note that *Foxf1* requires an additional (epithelial) signaling input to upregulate and confine it to the inner mesenchymal domain. SMO-mediated HH signaling also directs cell survival in the outer mesenchymal domain but this activity is independent of *Foxf1* and *Bmp4*.

Here, we provided genetic evidence that mesenchymal HH signaling controls proliferation and differentiation both in the inner region of the mesenchyme as well as in the ureteric epithelium, and that these programs are relayed by a FOXF1-BMP4 module. HH signaling also regulates survival of outer mesenchymal cells, which however employs other effector genes. We conclude that SHH is the crucial signal for coordinated tissue growth and cell differentiation in early ureter development (Fig 8).

### HH signaling controls different cellular programs in early ureter development

The functional significance of *Shh* for ureter development was initially addressed by a conditional gene targeting experiment. Mice with loss of *Shh* from the nephric duct and its derivatives showed reduced mesenchymal proliferation and delayed and reduced SMC differentiation, and developed hydroureter with associated hydronephrosis at birth [8]. Later, mice with a complete loss of *Shh* were reported to display with 50% penetrance bilateral renal aplasia or hydroureter and hydronephrosis indicating a more profound role for SHH in the development of the complete upper urinary tract [11, 40]. Targeted inactivation of HH signaling in the mesenchyme surrounding the renal pelvis and upper ureter did not compromise SMC differentiation but interfered with establishment of cells required for impulse initiation and propagation indicating an additional role for *Shh* in the development of ureter peristalsis [41].

Our study aimed to better understand the role of HH signaling in early ureter development by deleting its unique signaling mediator gene *Smo* in the ureteric mesenchyme. We observed reduced proliferation rates in the inner mesenchymal region at E12.5, a failure to initiate SMC differentiation at E14.5, and formation of hydroureter at birth. This confirms the previous studies that epithelial SHH is crucial for the structural architecture of the ureter by regulating in a paracrine fashion the proliferation and SMC differentiation of adjacent mesenchymal cells. The difference in the severity of SMC defects in the conditional loss-of-function models may derive from variable efficiency of cre recombination or different genetic backgrounds. Alternatively, it may indicate that other HH family members provide a minor but relevant input to SMO-mediated signaling in the ureter. A paradigm for this is found in the intestinal epithelium where SHH and IHH cooperatively signal to the underlying mesenchyme [42, 43].

In contrast to previous studies [8, 11], we also analyzed the consequence of abrogation of HH signaling for epithelial development. To our surprise, we found that proliferation and differentiation of the epithelial compartment was similarly affected than that of the mesenchyme; proliferation was reduced at E12.5 and epithelial differentiation was not initiated at E14.5 resulting in epithelial hypoplasia and a complete lack of urothelial cell types at E18.5. While it is possible that epithelial SHH uses paracrine and autocrine signaling pathways to regulate proliferation rates in the mesenchyme and epithelium, respectively, our approach to manipulate the signaling pathway in the mesenchyme disfavors an autocrine mode of HH signaling and rather suggests that mesenchymal HH signaling uses a relay signal to affect epithelial cell cycle progression and differentiation.

Since cell density and cell number can affect cellular differentiation programs [44], the argument may arise that epithelial and mesenchymal differentiation defects are secondary to the severe hypoplasia found in *Smo*-deficient ureters. However, our genetic rescue experiments showed that robust SMC and urothelial differentiation occurs despite massive tissue hypoplasia, indicating that HH controlled cyto-differentiation does not rely on cell number.

Misexpression of a constitutively active form of SMO in the entire ureteric mesenchyme resulted in increased cell proliferation in the inner mesenchymal region and the epithelium indicating that the level of SHH is a limiting factor in this program. However, it did not result in ectopic or enhanced SMC differentiation in the outer mesenchymal layer. Together with our recent finding that loss of canonical WNT signaling in the ureteric mesenchyme abrogates the SMC investment of the ureter [10], it seems plausible that initiation of SMC differentiation depends on the combinatorial input of both epithelial SHH and WNT signals and that the short range signaling activity of the latter is decisive to restrict the program to mesenchymal cells adjacent to the ureteric epithelium. WNT signals may also impinge on mesenchymal proliferation since proliferation rates were reduced but not stalled in mice with mesenchyme specific loss of both WNT or HH signaling in the ureter [10].

Our study also found that loss of HH signaling in the ureteric mesenchyme results in a massive increase of programmed cell death in the outer mesenchymal domain at E12.5, and severe ureter hypoplasia from E14.5 onwards. Moreover, expression of a conditionally activated form of SMO completely abolished apoptosis in the lateral mesenchymal domain at E11.5 and led to massive tissue hyperplasia of this region from which adventitial fibrocytes normally arise. Importantly, pharmacological activation of SMO was sufficient to trigger survival of isolated ureteric mesenchyme. These findings demonstrate that HH signaling is required and sufficient to maintain cell survival in the outer region of the undifferentiated ureteric mesenchyme. The tight spatial and temporal regulation of this activity may be instrumental in defining the size of adventitial precursor pool as well as severing the ureter from the kidney. In any case, the finding that HH signaling suffices to maintain ureteric mesenchymal cells *in vitro*, may open avenues for easier manipulation of these progenitors in the future.

All in all, our data suggest that epithelial SHH signals are crucial for the coordinated elongation of the early ureter tube by controlling the survival of tunica adventitia precursor cells and by coordinating proliferation and differentiation of the inner mesenchymal cell layer and the adjacent epithelial compartment.

### HH signaling employs a FOXF1-BMP4 module to mediate mesenchymal and epithelial proliferation and differentiation programs in the ureter

Previous work showed that expression of *Bmp4* in the ureteric mesenchyme depends on *Shh*, and that loss or antagonism of BMP4 leads to mesenchymal differentiation defects [8, 9, 45, 46]. However, it was unclear whether all aspects of HH signaling are mediated by BMP4 and how *Bmp4* expression is regulated. Our work suggests that FOXF1 is the crucial mesenchymal effector of HH signaling that exerts its function in proliferation and differentiation of the mesenchyme and epithelium through and in concert with BMP4.

Our microarray analysis identified the three Forkhead transcription factor genes *Foxf1*, *Foxl1* and *Foxf2* to strongly depend in their expression in the ureter on HH signaling. *In situ* hybridization analysis detected expression of *Foxf1* but not of *Foxl1* and *Foxf2* in the ureteric mesenchyme suggesting that the latter two factors play a minor role in ureter development. Intriguingly, *Foxf1* expression was upregulated at E14.5 in the inner layer of mesenchymal cells, i.e. shortly before the onset of SMC and epithelial differentiation. This contrasts with the activity of HH signaling which is present from at least E11.5 onwards in the inner and outer layers of the ureteric mesenchyme [8]. Together with the observation that ectopic HH signaling (i.e. activated SMO) is not sufficient to induce SMC differentiation and *Foxf1* in the outer mesenchymal region, this argues that *Foxf1* expression requires a critical second input, possibly WNTs as discussed above, from the epithelial compartment around E13.5. Alternatively or in parallel, a mesenchymal activity may counteract HH activation of *Foxf1* expression until that stage.

We used conditional misexpression of a variant of FOXF1 encoding a strong transcriptional repressor to address the function of this transcription factor in the ureteric mesenchyme. Such an approach is not without risk since it may interfere with activity of other family members in early ureter development. However, of the more than 40 members of the Fox family of transcription factors, expression of only a few have been identified in ureter development including *Foxd1* in tunica adventitia cells, *Foxc1* and *Foxc2* in the early ureteric mesenchyme, and *Foxa1* in the ureteric epithelium [4, 47, 48]. Importantly, FOXF1 and FOXF2 represent an evolutionary conserved but isolated subgroup of FOX proteins, and FOX transcription factors show a high divergence of binding sites [49, 50] arguing that FOXF1-DN only interferes with FOXF1 and FOXF2 function. Importantly, the defects we see do not phenocopy loss of any known Forkhead gene in the ureter but recapitulate the proliferation and differentiation defects of *Smo* loss-of-function mutants in the inner mesenchymal domain and the epithelium of the developing ureter. Moreover, mesenchymal expression of FOXF1 partially ameliorated tissue hypoplasia and completely rescued the mesenchymal and epithelial differentiation defects in cyclopamine treated ureters arguing together that FOXF1 is the crucial and unique mesenchymal mediator of inputs from SHH and other epithelial signals in the control of these cellular programs.

While it is conceivable that FOXF1 directly activates the SMC program in the ureteric mesenchyme, its epithelial functions must be mediated by a diffusible factor. Our molecular analyses have shown that expression of *Bmp4* in the ureteric mesenchyme strictly depends on HH signaling and FOXF1 activity in this tissue. BMP4 was sufficient to rescue the proliferation defects in both tissue compartments as well as to induce epithelial differentiation in ureter explant cultures in which HH signaling or FOXF1 function was abrogated. BMP4 did not rescue SMC differentiation in these settings arguing that FOXF1 acts upstream of and in concert with BMP4 in this program. While this study was in progress, we showed that loss of *Bmp4* in the ureteric mesenchyme resulted in a complete loss of epithelial and mesenchymal proliferation and differentiation further supporting the notion that BMP4 mediates HH signaling and FOXF1 activity in the ureteric mesenchyme [51].

It is important to note that SHH, FOXF1 and BMP4 constitute a regulatory axis that has been adopted in other developmental contexts, including the morphogenesis of the developing gastrointestinal tract [30, 31], the establishment of left/right asymmetry in the lateral plate mesoderm [52] and vasculogenesis of the yolk sac [53]. While the presence of GLI1 binding sites in the regulatory regions of *Foxf1* (and *Foxl1*) characterized this gene as a direct target of HH signaling [31, 54, 55], it remains to be seen whether *Bmp4* is directly controlled by FOXF1 transcriptional activity in the ureter or in any of these contexts.

Our functional experiments provided compelling evidence that FOXF1 and BMP4 mediate the proliferation and differentiation function of HH signaling in the ureter, but it seems unlikely that these factors also account for the anti-apoptotic activity of this pathway in the outer mesenchymal domain. First, *Foxf1*, *Foxf2* and *Foxl1* were not detectably expressed in the ureteric mesenchyme at E11.5 to E12.5 when this HH activity occurs. Second, apoptosis of outer mesenchymal cells was not detected in mutants with expression of the repressor version of FOXF1 in the ureteric mesenchyme. Third, FOXF1 was not sufficient to rescue tissue hypoplasia after abrogation of HH signaling. Fourth, loss of *Bmp4* in the ureteric mesenchyme does not affect cell survival in adventitial precursor cells of the ureter [51]. Finally, BMP4 antagonism did not abolish the puromorphamine induced hyperplasia of outer mesenchymal cells in ureter explants. In other developmental contexts (e.g. the spinal cord) it was shown that HH signaling directly regulates the expression of the anti-apoptotic gene *Bcl2* [56]. Our microarray analysis showed that *Bcl2* is slightly reduced upon cyclopamine treatment of ureter explants (S2 Table) making it a possible contributor of the pro-survival role of HH signaling. We detected strong reduction of expression of *Ddit4l* whose homologue DDIT4 in at least some biological contexts was reported to exert an anti-apoptotic function [57, 58] making it another candidate for this function.

### SHH-FOXF1-BMP4 and human congenital urinary tract anomalies

Given the requirement of the SHH-FOXF1-BMP4 regulatory axis for SMC differentiation in the ureter and the severity of the hydroureter formation associated with their complete or partial loss in the mouse, it is obvious that the components of this axis represent candidate genes for human congenital anomalies of the kidney and urinary tract (CAKUT). In fact, heterozygous loss-of-function mutations in *BMP4* and *GLI* transcription factor genes have been identified in patients with ureter anomalies, and hypo- and dysplastic kidneys [59, 60]. Since these genes are expressed and required in numerous embryonic programs, renal defects are often associated with a whole spectrum of other organ malformations.

Interestingly, heterozygous inactivation of *FOXF1* has been associated with the “Alveolar Capillary Dysplasia with Misalignment of Pulmonary Veins” syndrome (ACDMPV: OMIM 265380) in human newborns [61, 62]. This syndrome is characterized by failure of formation and ingrowth of alveolar capillaries and anomalously situated pulmonary veins. Affected infants present with respiratory distress resulting from pulmonary hypertension in the early postnatal period, and the disease is fatal within the newborn period [63, 64]. Additional defects occur in the cardiovascular, gastrointestinal, musculoskeletal systems and in the urinary tract. In the latter vesico-ureteric and pelvic-ureteric junction obstructions, hydroureter and hydronephrosis have been reported [65], pointing to haploinsufficiency of *FOXF1* for ureter development.

Previous work tried to find whether mutations in FOXF1 are also associated with the VATER/VACTERL combination of congenital anomalies that includes vertebral defects, anorectal malformations, cardiac defects, tracheoesophageal fistula with or without esophageal atresia, renal malformations, and limb defects. Targeted sequencing in 123 patients with VATER/VACTERL or VATER/VACTERL-like phenotype detected a *FOXF1 de novo* mutation in one patient. *In situ* hybridization analyses in mouse embryos identified *Foxf1* expression in the development of most VATER/VACTERL organ systems except the urinary tract [66], questioning the significance of mutations in *FOXF1* for renal disease manifestations [67]. With our finding that *Foxf1* is expressed and functionally required in ureter development in the mouse, the gene is (back) on the list of candidates for forms of CAKUT with additional extra-renal disease manifestations in human newborns.

## Materials and methods

### Ethics statement

All animal experiments were performed in compliance with the German animal protection law, Tierschutzgesetz (TierSchG, BGBl. I S. 1206, 1313, 2006/05/18). All mice were housed and handled according to good animal practice as defined by FELASA (Federation of European Laboratory Animal Science Associations) and the national animal welfare body GV-SOLAS (Gesellschaft fur Versuchstierkunde/Society for Laboratory Animal Science). All animal experiments were approved by the Lower Saxony Committee on the Ethics of Animal Experiments as well as the responsible state office (Lower Saxony State Office of Consumer Protection and Food Safety) under the permit numbers 33.12-42502-04-13/1356 and AZ33.14-42502-04-13/1264.

### Generation of *Hprt*^*Foxf1*^ and *Hprt*^*Foxf1DN*^ alleles

The *Foxf1* open reading frame was PCR-amplified from cDNA with primer pairs Foxf1-for (*Nhe*I) ATG CAC TAG TAT GTC CGC GCC CGA CAA GC and Foxf1-rev (*Nde*I) ATG CCA TAT GTC ACA TCA CAC ACG GCT TGA TG or Foxf1ΔStop-rev (*Nde*I) ATG CCA TAT GCA TCA CAC ACG GCT TGA TG with the latter introducing a mutated stop codon. A DNA fragment encoding the engrailed repressor domain (ENG) was PCR-amplified from the *pCS2*^+^.*Engrailed* plasmid [35] with primer pairs Eng-for (*Nde*I) GAG ACA TAT GGC CCT GGA GGA TCG C and Eng+Stop-rev (*Nde*I) GAG ACA TAT GCT AGA GGC TCG AGA GGG ATC C. Restriction sites introduced via primers were used to insert PCR products into *Nhe*I-*Nde*I sites of a shuttle vector containing *IRES-GFP* to generate *FOXF1-IRES-GFP* (for *Hprt*^*Foxf1*^) and *FOXF1ENG-IRES-GFP* (for *Hprt*^*Foxf1DN*^) constructs. These constructs were then subcloned into the *pMP8*.*CAG-Stop* vector [68] using restriction enzymes *Swa*I and *Mlu*I. To target the *Hprt* locus, linearized constructs were electroporated into E14TG2a embryonic stem cells that carry a deficient *Hprt* locus enabling HAT selection after correct targeting and restoration of the locus [36, 37]. Correctly targeted ES cell clones were selected with HAT medium (Gibco), expanded and genotyped by PCR. Verified ES clones were microinjected into CD1 mouse blastocysts. Chimeric males were obtained and mated to NMRI females to produce heterozygous F1 females.

### Mouse strains and husbandry

*Smo*^*tm2Amc*^ *(synonym*: *Smo*^*fl*^) [14], *Gt(ROSA)26Sor*^*tm1(Smo/EYFP)Amc*^ (synonym: *R26*^*SmoM2*^) [17], *Gt(ROSA)26Sor*^*tm4(ACTB-tdTomato-EGFP)Luo*^ (synonym: *R26*^*mTmG*^) [19 and Axin2tm1(cre/ERT2)Rnu (synonym: Axin2creERT2) {van Amerongen, 2012 #47] mouse lines were all obtained from the Jackson Lab. The *Tbx18*^*tm4(cre)Akis*^ (synonym: *Tbx18*^*cre*^) mouse line was previously generated in the lab [69]. All lines were maintained on an NMRI outbred background. *Tbx18*^*cre/+*^;*Smo*^*fl/fl*^ (synonym: *Smo*^*LOF*^) embryos were obtained from matings of *Tbx18*^*cre/+*^;*Smo*^*fl/+*^ males and *Smo*^*fl/fl*^ females. *Tbx18*^*cre/+*^;*R26*^*mTmG/SmoM2*^ (synonym: *Smo*^*GOF*^) and *Tbx18*^*cre/+*^;*R26*^*mTmG/+*^ embryos were derived from matings of *Tbx18*^*cre/+*^;*R26*^*mTmG/mTmG*^ males and *R26*^*SmoM2/SmoM2*^ and NMRI females, respectively. *Tbx18*^*cre/+*^;*Hprt*^*Foxf1DN/y*^ (synonym: *Foxf1DN*) and *Tbx18*^*cre/+*^;*Hprt*^*Foxf1/y*^ embryos were obtained from matings of *Tbx18*^*cre/+*^ males with *Hprt*^*Foxf1DN/Foxf1DN*^ and *Hprt*^*Foxf1/Foxf1*^ females, respectively. *Axin2*^*creERT2/+*^;*Hprt*^*Foxf1/y*^ embryos were obtained from matings of *Axin2*^*creERT2/+*^ males with *Hprt*^*Foxf1/Foxf1*^ females, respectively. For all matings cre negative littermates were used as controls. Mouse strains, genotypes, conditions and numbers for each experiment are summarized in S1 Table. For timed pregnancies, vaginal plugs were checked in the morning after mating, and noon was defined as embryonic day (E) 0.5. Embryos and urogenital systems were dissected in PBS. Ureters for explant cultures were dissected in L-15 Leibovitz medium (Biochrom). Specimens were fixed in 4% PFA/PBS, transferred to methanol and stored at -20°C prior to immunofluorescence or *in situ* hybridization analyses. PCR genotyping was performed on genomic DNA prepared from yolk sac or tail biopsies. All mice were bred and maintained in the central animal facility of the Medizinische Hochschule Hannover (Hannover, Germany) according to institutional guidelines. All experiments were performed with approval of the authorities of the State of Lower Saxony.

### Histological and expression analyses

Embryos, urogenital systems and ureters were paraffin-embedded and sectioned to 5 μm. Hematoxylin and eosin staining was performed according to standard procedures.

Non-radioactive *in situ* hybridization analysis of gene expression was performed on whole-mount specimens or on 10-μm paraffin sections of the proximal ureter with digoxigenin-labeled antisense riboprobes [70, 71].

For immunofluorescence analysis on 5-μm paraffin sections polyclonal rabbit-anti-TAGLN (1:250, ab14106, Abcam), monoclonal mouse-anti-GFP (1:250, 11814460001, Roche), monoclonal mouse-anti-ACTA2 (1:250, A5228, Sigma-Aldrich), polyclonal rabbit-anti-ΔNP63 (1:250, 619001, Biolegend), polyclonal rabbit-anti-KRT5 (1:250, PRB-160P, Covance), monoclonal mouse-anti-UPK1B (1:250, WH0007348M2, Sigma-Aldrich), monoclonal mouse-anti-BrdU (1:250, 1170376, Roche) or polyclonal rabbit antisera against CDH1 (1:250, gift from Rolf Kemler) and MYH11 (1:250, gift from Robert Adelstein) were used as primary antibodies. Biotinylated goat-anti-rabbit IgG (1:250, 111065033, Dianova), Alexa488-conjugated goat-anti-rabbit IgG (1:500, A11034, Molecular Probes) and Alexa555-conjugated goat-anti-mouse IgG (1:500, A21422, Molecular Probes) were used as secondary antibodies. The signal of the ΔNP63 antibody was amplified using the Tyramide Signal Amplification (TSA) system (NEL702001KT, Perkin Elmer). Before staining, paraffin sections were deparaffinized and cooked for 15 min in antigen unmasking solution (H-3300, Vector Laboratories). Nuclei were stained with 4',6-diamidino-2-phenylindole (DAPI). At least three specimens of each genotype were used for each of these analyses.

### Cellular assays

Cell proliferation rates were analyzed by the detection of incorporated BrdU on 5 μm paraffin sections according to published protocols [72]. A minimum of 12 sections of the proximal ureter from 3 independent specimens was analyzed per genotype. The BrdU-labeling index was defined as the number of BrdU-positive nuclei relative to the total number of nuclei as detected by DAPI counterstaining in arbitrarily defined compartments of the ureter. Data were expressed as mean ± standard deviation. The two-tailed Student’s t-test was used to test for significance. P≤0.05 was regarded as significant, P≤0.005 as highly significant and P≤0.001 as extremely significant.

Apoptosis was analyzed on 5 μm paraffin sections using the ApopTag Plus Fluorescein *In Situ* Apoptosis Detection Kit (Chemicon). Alternatively, LysoTracker Red DND-99 (Thermo Scientific) was used to detect cell death in organ cultures. Briefly, E11.5 kidney rudiments were cultured for 1 d and incubated for 1 h with 2.5 μM LysoTracker prior to documentation.

### Organ cultures

Kidney rudiments or ureters were dissected from the embryo, explanted on 0.4 μm polyester membrane Transwell supports (Corning) and cultured at the air-medium interface with DMEM/F12 (Gibco) supplemented with 10% FCS (Biochrom), and 1% of concentrated stocks of Penicillin/Streptomycin, Pyruvate and Glutamax (Gibco). For pharmacological manipulation of SHH signaling cyclopamine (Selleckchem) and purmorphamine (Millipore) were used at a final concentration of 10 μM and 2 μM, respectively. Recombinant mouse BMP4 (R&D Systems) was used at a final concentration of 100 ng/ml. BMP4 inhibitor NOGGIN (ABIN2018288, antikoerper-online.de) was dissolved in water to 10 μg/ml. To induce recombination with the *Axin2*^*creERT2*^ line 4-Hydroxytamoxifen (H7904, Sigma-Aldrich) was added to the medium at a final concentration of 500 nM for the first 24 h of culture. Culture medium was replaced every day. Contralateral kidneys/ureters were used in control groups.

### Microarray

Two independent pools of 50 E12.5 left and right ureters were cultured with DMSO or 10 μM cyclopamine for 18 h. Total RNA was extracted with peqGOLD RNApure (PeqLab) and was sent to the Research Core Unit Transcriptomics of Hannover Medical School where RNA was Cy3-labeled and hybridized to Agilent Whole Mouse Genome Oligo v2 (4x44K) Microarrays. To identify differentially expressed genes, normalized expression data was filtered using Excel based on an intensity threshold of 150 and a more than 2 fold change in both pools.

### Image analysis

Sections and organ cultures were photographed using a Leica DM5000 microscope with Leica DFC300FX digital camera or a Leica DM6000 microscope with Leica DFC350FX digital camera. Urogenital systems were documented using a Leica M420 microscope with a Fujix HC-300Z digital camera. Figures were prepared with Adobe Photoshop CS4.

## Supporting information

S1 Fig The cellular composition of the developing and mature ureter. (Co-) Immunofluorescence analysis of expression of the SMC marker TAGLN and the lineage marker GFP and of the epithelial markers ΔNP63, KRT5 and UPK1B on transverse sections of the proximal ureter in *Tbx18*^*cre/+*^;*R26*^*mTmG/+*^ mice at E18.5 and in adults at P40. Note that *Tbx18*^*cre*^ specifically mediates recombination in the ureteric mesenchyme and that, thus, the GFP reporter labels these cells. Nuclei are counterstained with DAPI. The diagram shows the cellular composition of the ureter at E18.5 and in adults at P40 as defined by expression of these markers. In the mesenchymal wall, *Tunica adventitia* fibrocytes are GFP^+^TAGLN^-^ outer cells, SMCs are GFP^+^TAGLN^+^ medial cells, and *Lamina propria* fibrocytes are GFP^+^TAGLN^-^ inner cells. In the epithelial compartment, the basal layer is composed of ΔNP63^+^KRT5^+^UPK1B^-^ cells, the intermediate layer of ΔNP63^+^KRT5^-^UPK1B^+^ cells, and the superficial layer of ΔNP63^-^KRT5^-^UPK1B^+^ cells.
(TIF)

S2 Fig 10 μM Cyclopamine is sufficient to largely inhibit HH signaling in ureter explant cultures. Wildtype ureters were isolated at E12.5, cultured for 18 h in the presence of DMSO or 10 μM cyclopamine (Cyc) and subjected to *in situ* hybridization analysis of expression of the SHH target gene *Ptch1*. Reduced expression of *Ptch1* in the cyclopamine treated culture indicates that HH signaling is severely compromised under these conditions.
(TIF)

S3 Fig HH signaling acts at E11.5 to E13.5 in ureter development. Wildtype ureters were isolated at E11.5 and cultured for 8 days in the presence of DMSO or 10 μM Cyclopamine in intervals of 2 days (D) as indicated. Immunofluorescence of the SMC marker ACTA2 (in red) in presence of the epithelial counterstain CDH1 (in green) shows that only treatment of the ureter explants at day 1 and 2 of the culture abrogates SMC differentiation in the ureter.
(TIF)

S4 Fig *In situ* hybridization analysis of genes downregulated in microarrays of cyclopamine treated ureters. Note that specific expression of *Foxl1*, *Foxf2*, *Crym*, *Ndp* and *Wif* was not detected in the ureter of wildtype, *Tbx18*^*cre/+*^;*Smo*^*fl/fl*^ (*Smo*^*LOF*^) and *Tbx18*^*cre/+*^;*R26*^*mTmG/SmoM2*^ (*Smo*^*GOF*^) embryos at E12.5 and E14.5.
(TIF)

S5 Fig Expression analysis of genes important for SMC differentiation in ureters with loss- and gain-of-function of HH signaling in the ureteric mesenchyme. (A) Table of genes. Shown are average intensities of transcripts in control and cyclopamine treated ureters and average fold changes (FC) of RNA intensities between the pools in two independent microarray experiments. (B) RNA *in situ* hybridization analysis on transverse sections of the proximal ureter region of control, *Smo*^*LOF*^ and *Smo*^*GOF*^ embryos at E12.5, and of control and *Smo*^*LOF*^ embryos at E14.5. Note that *Bmp4* and *Tcf21* expression is reduced in *Smo*^*LOF*^ ureters, and increased in *Smo*^*GOF*^ ureters at E12.5. Expression of *Tbx18*, *Tshz3*, *Sox9* and *Axin2* appears unaffected by loss- and gain-of-HH signaling in the ureteric mesenchyme.
(TIF)

S6 Fig Molecular analysis of marker gene expression in control and *Tbx18*^*cre/+*^;*Hprt*^*Foxf1DN/y*^ (*Foxf1DN*) ureters at E12.5 and E14.5. *In situ* hybridization on proximal ureter sections shows that markers of the inner domain of the ureteric mesenchyme, *Axin2*, *Tshz3*, *Tcf21*, *Tbx18* and *Sox9* are not changed in their expression in *Foxf1DN* ureters.
(TIF)

S7 Fig FOXF1 induces expression of *Bmp4* in the ureteric mesenchyme. Analysis of proximal sections of ureters explanted from E12.5 wildtype and *Axin2*^*creERT2/+*^;*Hprt*^*Foxf1/y*^ embryos and cultured for 3 d in the presence or absence of 10 μM cyclopamine or DMSO solvent by *in situ* hybridization for expression of *Foxf1* and *Bmp4*. Abrogation of HH signaling by cyclopamine leads to loss of *Foxf1* and *Bmp4* expression in the ureteric mesenchyme. *Axin2*^*creERT2*/+^ mediated recombination of the *Hprt*^*Foxf1*^ allele results in robust expression of *Foxf1* and induction of *Bmp*4 showing that FOXF1 is required and sufficient for *Bmp4* expression in the ureteric mesenchyme.
(TIF)

S1 Table Summary of genotypes, conditions and number of specimens used for each experiment. (PDF)

S2 Table List of transcripts identified by microarray analysis as dependent in their expression on HH signaling in the developing ureter. Shown are two lists of transcripts with reduced and enhanced expression after an 18-h treatment of E12.5 ureter explants with 10 μM cyclopamine. Two groups each of untreated and cyclopamine-treated ureters are shown with their intensities, and the resulting fold changes in expression upon comparison. For genes with reduced intensities after cyclopamine treatment, the intensity threshold was 200 for the control; fold changes were smaller than -1.4. For genes with enhanced intensities after cyclopamine treatment the intensity threshold was 200 for the treated group; fold changes were larger than 1.4.
(PDF)
