# Supplementary figures and images for: A SHH-FOXF1-BMP4 signaling axis regulating growth and differentiation of epithelial and mesenchymal tissues in ureter development
Source: PLoS Genet. 2017 Aug 10;13(8):e1006951. doi: 10.1371/journal.pgen.1006951 (PMC5567910; doi:10.1371/journal.pgen.1006951)

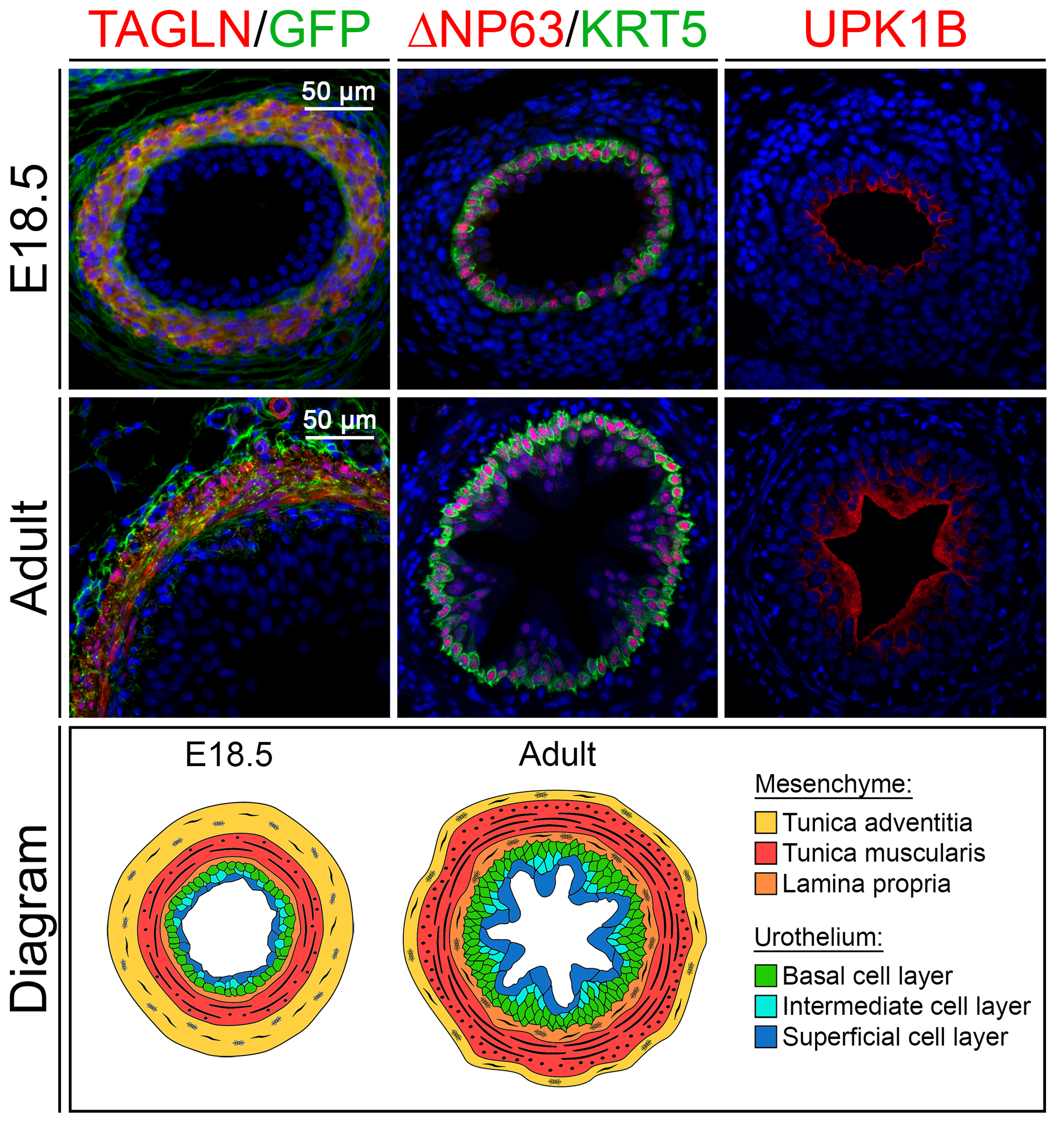

Supplement: S1 Fig — (Co-) Immunofluorescence analysis of expression of the SMC marker TAGLN and the lineage marker GFP and of the epithelial markers ΔNP63, KRT5 and UPK1B on transverse sections of the proximal ureter in Tbx18cre/+;R26mTmG/+ mice at E18.5 and in adults at P40. Note that Tbx18cre specifically mediates recombination in the ureteric mesenchyme and that, thus, the GFP reporter labels these cells. Nuclei are counterstained with DAPI. The diagram shows the cellular composition of the ureter at E18.5 and in adults at P40 as defined by expression of these markers. In the mesenchymal wall, Tunica adventitia fibrocytes are GFP+TAGLN- outer cells, SMCs are GFP+TAGLN+ medial cells, and Lamina propria fibrocytes are GFP+TAGLN- inner cells. In the epithelial compartment, the basal layer is composed of ΔNP63+KRT5+UPK1B- cells, the intermediate layer of ΔNP63+KRT5-UPK1B+ cells, and the superficial layer of ΔNP63-KRT5-UPK1B+ cells. (TIF) [file pgen.1006951.s001.tif]

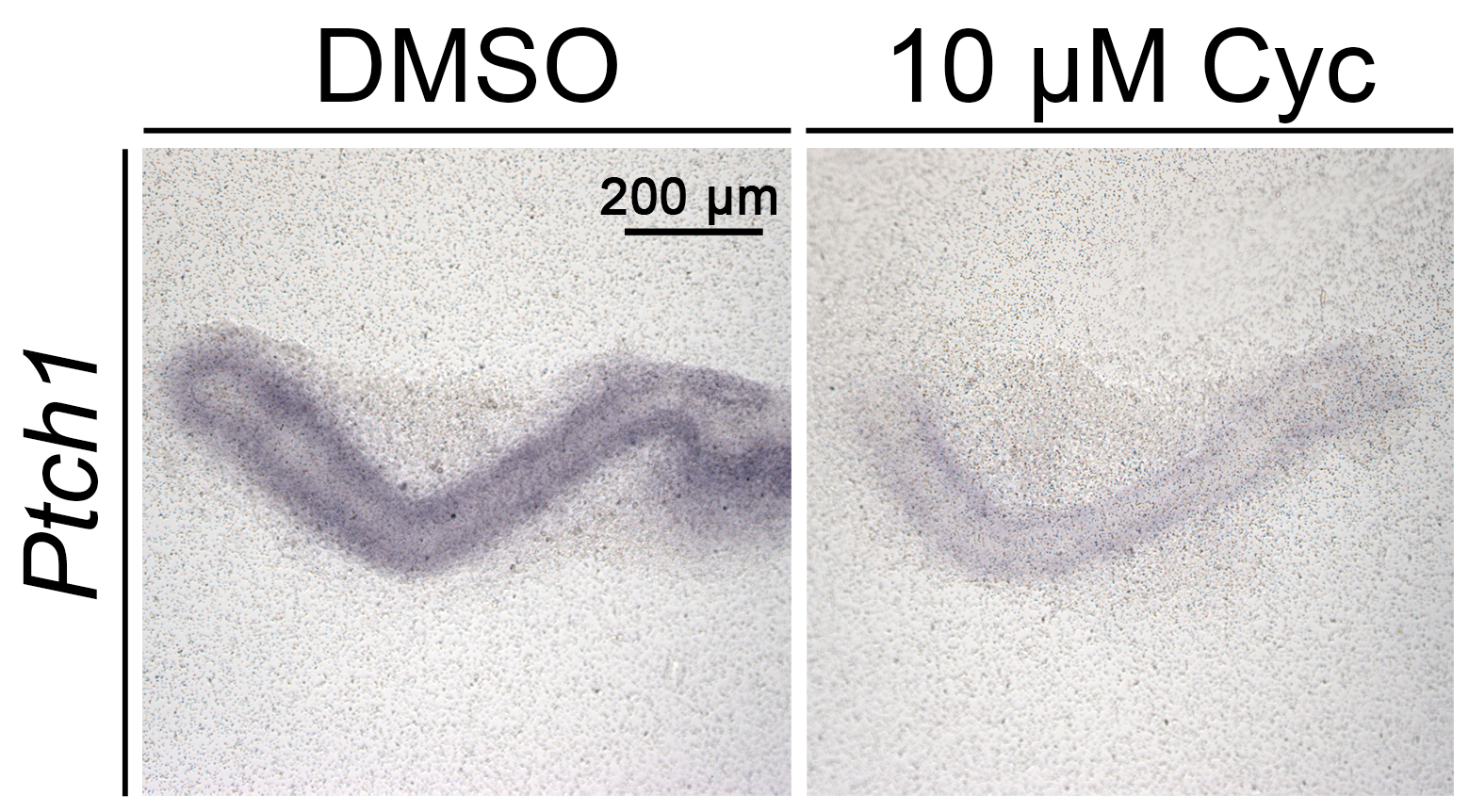

Supplement: S2 Fig — Wildtype ureters were isolated at E12.5, cultured for 18 h in the presence of DMSO or 10 μM cyclopamine (Cyc) and subjected to in situ hybridization analysis of expression of the SHH target gene Ptch1. Reduced expression of Ptch1 in the cyclopamine treated culture indicates that HH signaling is severely compromised under these conditions. (TIF) [file pgen.1006951.s002.tif]

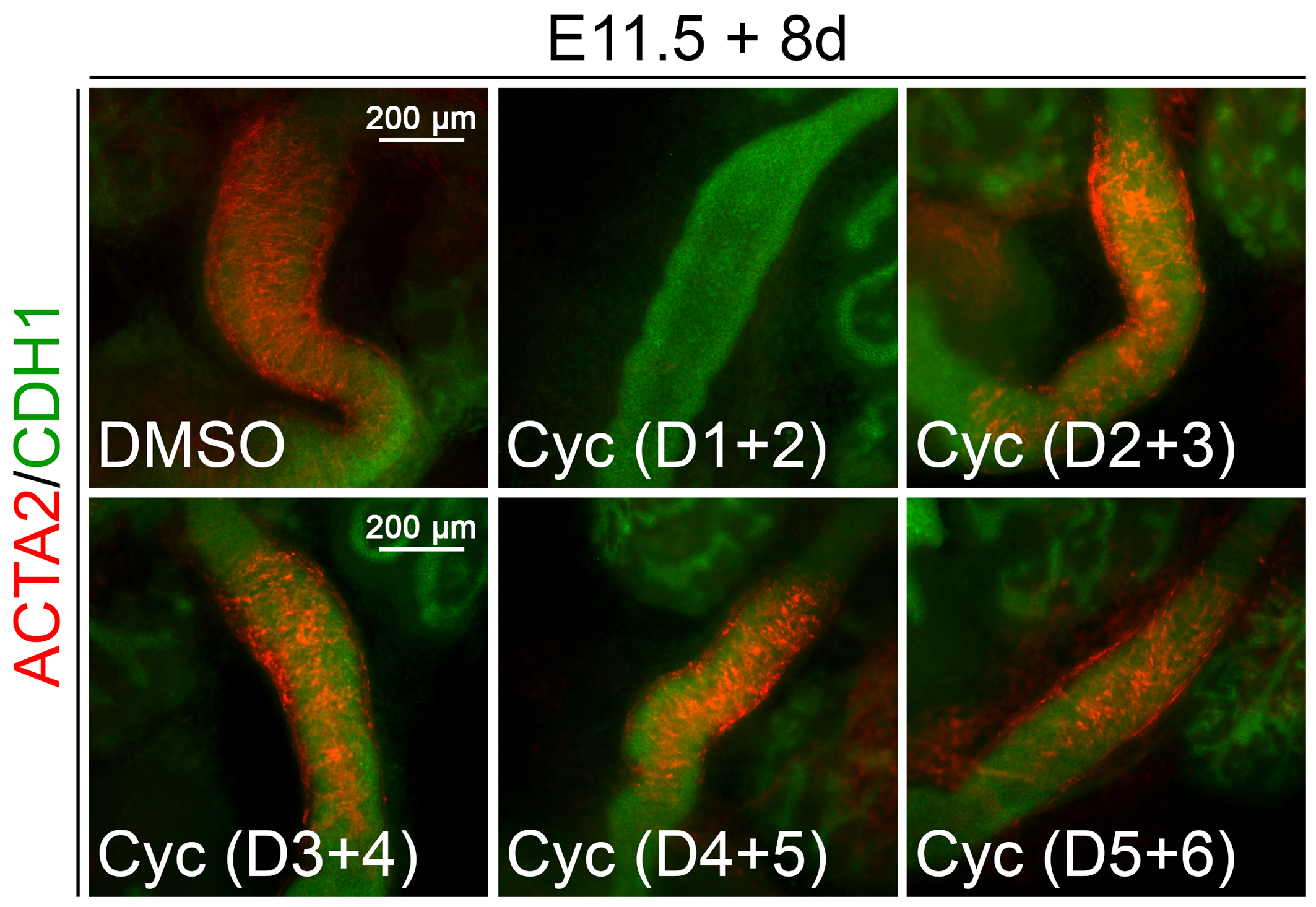

Supplement: S3 Fig — Wildtype ureters were isolated at E11.5 and cultured for 8 days in the presence of DMSO or 10 μM Cyclopamine in intervals of 2 days (D) as indicated. Immunofluorescence of the SMC marker ACTA2 (in red) in presence of the epithelial counterstain CDH1 (in green) shows that only treatment of the ureter explants at day 1 and 2 of the culture abrogates SMC differentiation in the ureter. (TIF) [file pgen.1006951.s003.tif]

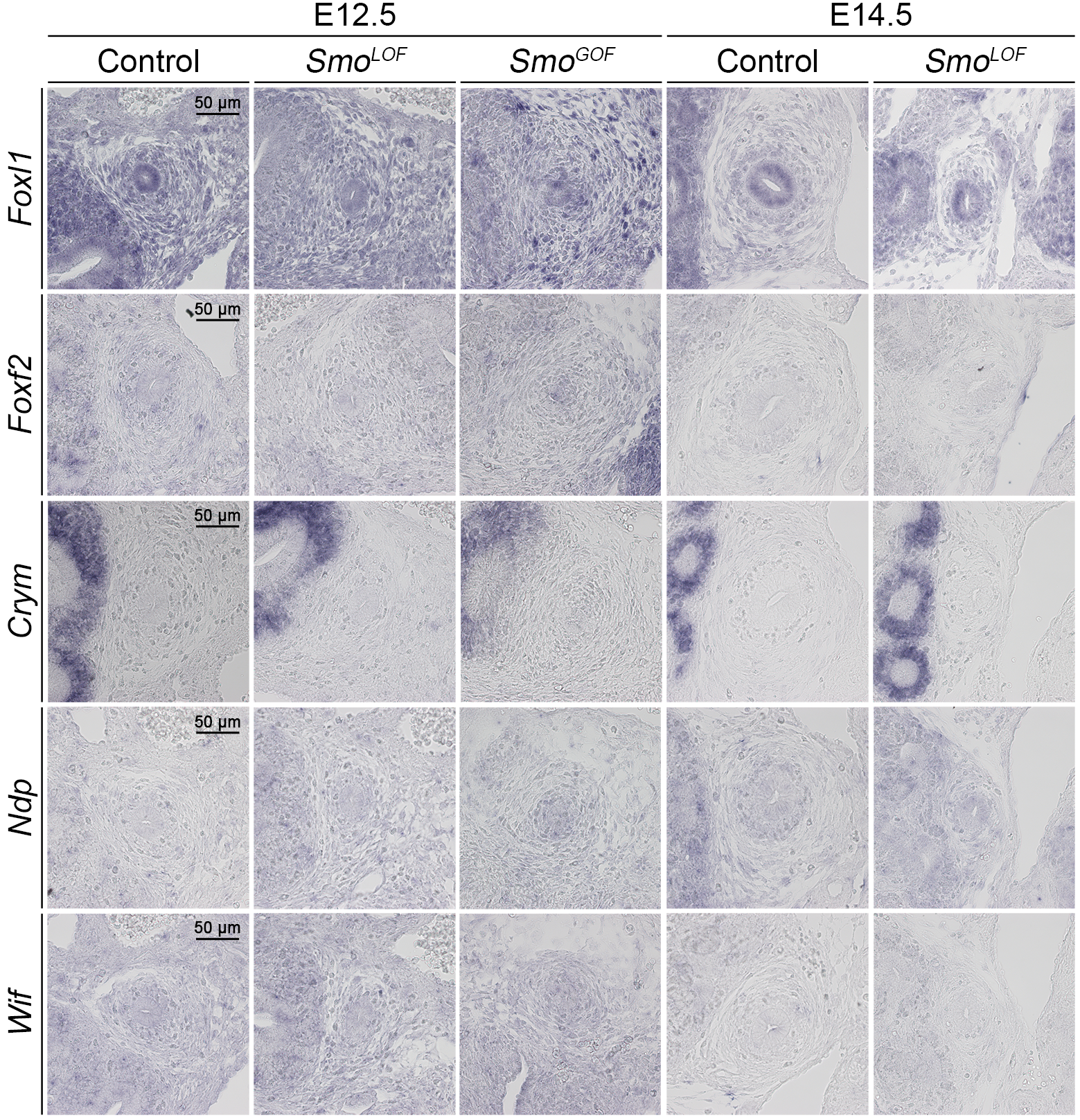

Supplement: S4 Fig — Note that specific expression of Foxl1, Foxf2, Crym, Ndp and Wif was not detected in the ureter of wildtype, Tbx18cre/+;Smofl/fl (SmoLOF) and Tbx18cre/+;R26mTmG/SmoM2 (SmoGOF) embryos at E12.5 and E14.5. (TIF) [file pgen.1006951.s004.tif]

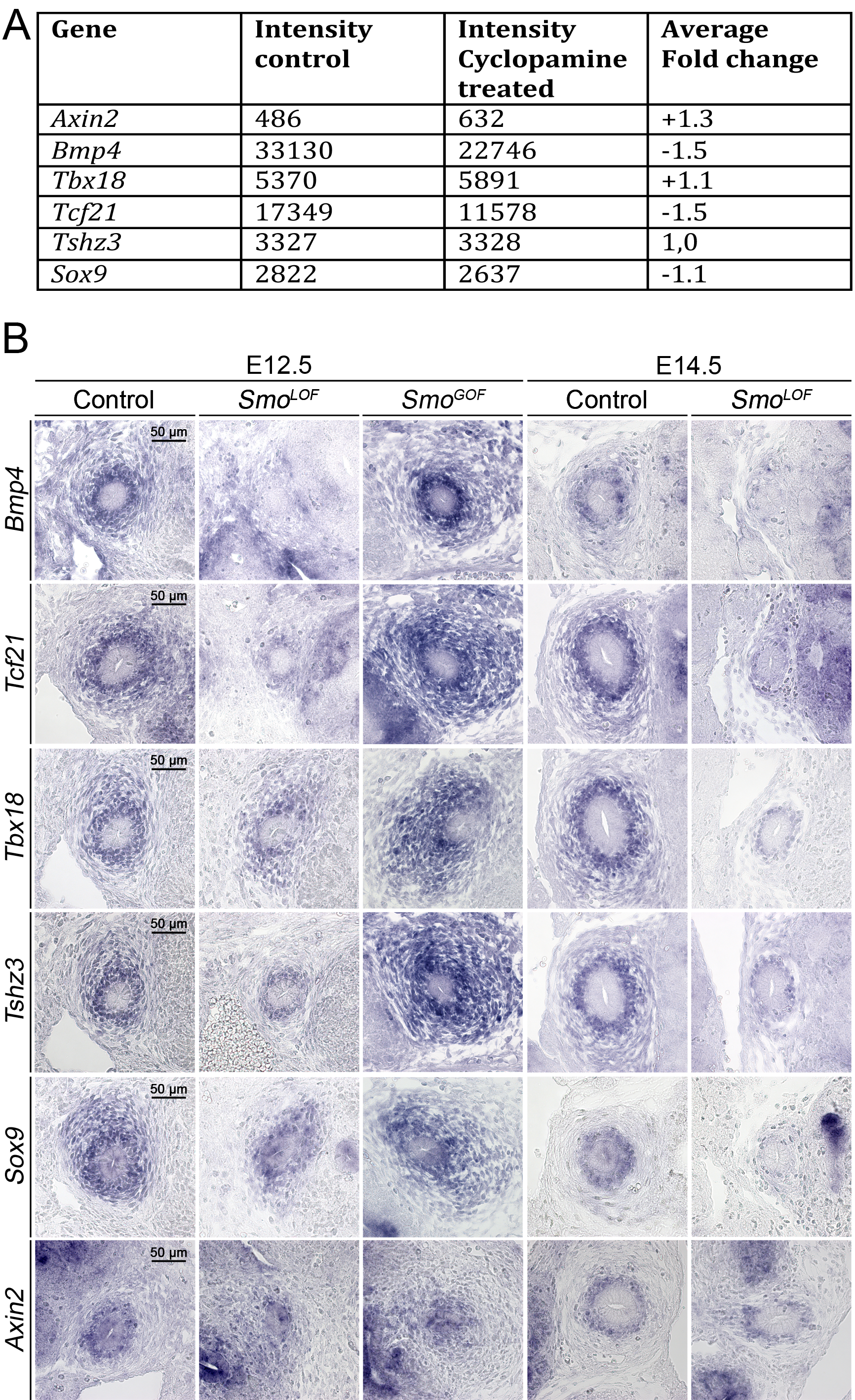

Supplement: S5 Fig — (A) Table of genes. Shown are average intensities of transcripts in control and cyclopamine treated ureters and average fold changes (FC) of RNA intensities between the pools in two independent microarray experiments. (B) RNA in situ hybridization analysis on transverse sections of the proximal ureter region of control, SmoLOF and SmoGOF embryos at E12.5, and of control and SmoLOF embryos at E14.5. Note that Bmp4 and Tcf21 expression is reduced in SmoLOF ureters, and increased in SmoGOF ureters at E12.5. Expression of Tbx18, Tshz3, Sox9 and Axin2 appears unaffected by loss- and gain-of-HH signaling in the ureteric mesenchyme. (TIF) [file pgen.1006951.s005.tif]

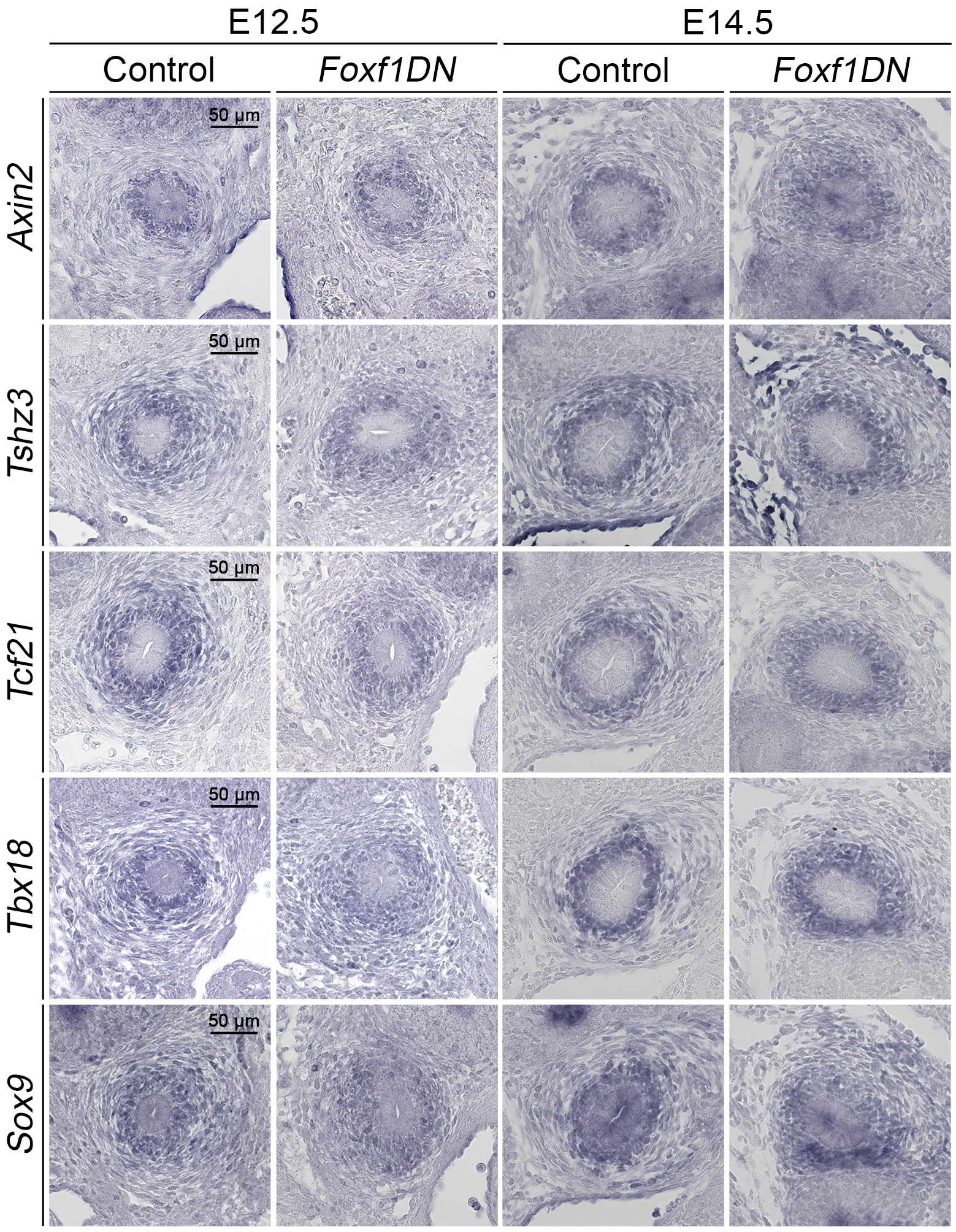

Supplement: S6 Fig — In situ hybridization on proximal ureter sections shows that markers of the inner domain of the ureteric mesenchyme, Axin2, Tshz3, Tcf21, Tbx18 and Sox9 are not changed in their expression in Foxf1DN ureters. (TIF) [file pgen.1006951.s006.tif]

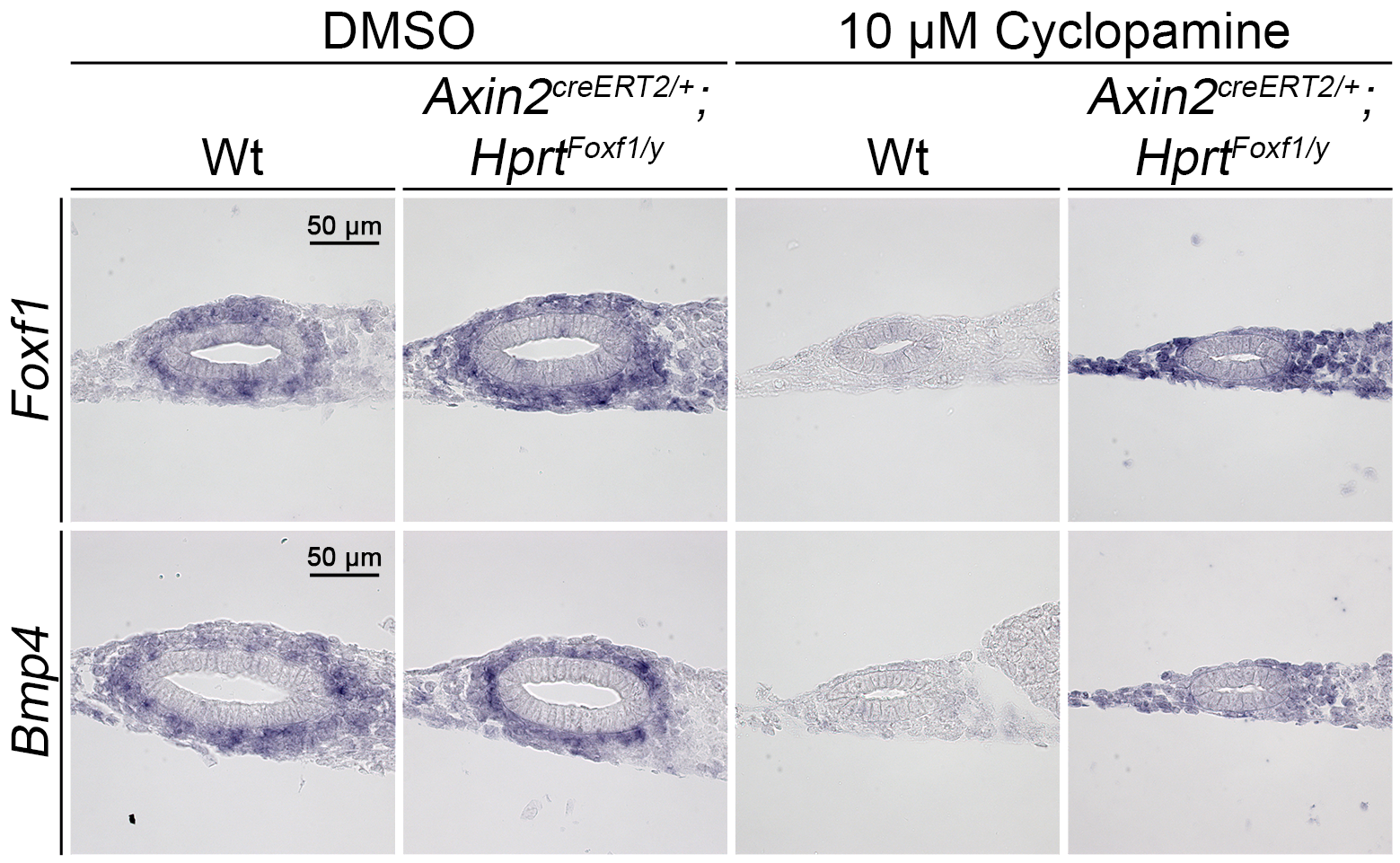

Supplement: S7 Fig — Analysis of proximal sections of ureters explanted from E12.5 wildtype and Axin2creERT2/+;HprtFoxf1/y embryos and cultured for 3 d in the presence or absence of 10 μM cyclopamine or DMSO solvent by in situ hybridization for expression of Foxf1 and Bmp4. Abrogation of HH signaling by cyclopamine leads to loss of Foxf1 and Bmp4 expression in the ureteric mesenchyme. Axin2creERT2/+ mediated recombination of the HprtFoxf1 allele results in robust expression of Foxf1 and induction of Bmp4 showing that FOXF1 is required and sufficient for Bmp4 expression in the ureteric mesenchyme. (TIF) [file pgen.1006951.s007.tif]
