# Supplementary material for: A SHH-FOXF1-BMP4 signaling axis regulating growth and differentiation of epithelial and mesenchymal tissues in ureter development
Source: PLoS Genet. 2017 Aug 10;13(8):e1006951. doi: 10.1371/journal.pgen.1006951 (PMC5567910; doi:10.1371/journal.pgen.1006951)
Supplement: S1 Table — (PDF) [file pgen.1006951.s008.pdf]

Figure	Stage	Specimens per genotype or condition	Genotypes / Conditions	Description of the experiment
1 A-D	E18.5	9	Tbx18cre/+; SmoLOF/+ (Control), Tbx18cre/+; SmoLOF/LOF (SmoLOF)	Morphological characterization of urogenital systems
1 E-F	E18.5	3	Tbx18cre/+; SmoLOF/+ (Control), Tbx18cre/+; SmoLOF/LOF (SmoLOF)	Histological characterization of the kidney
1 G-H	E18.5	3	Tbx18cre/+; SmoLOF/+ (Control), Tbx18cre/+; SmoLOF/LOF (SmoLOF)	Histological characterization of the proximal ureter
1 I-T	E18.5	3	Tbx18cre/+; SmoLOF/+ (Control), Tbx18cre/+; SmoLOF/LOF (SmoLOF)	Immunofluorescence and in situ hybridization analysis of ureteric differentiation
1 U-V	E18.5	3	Tbx18cre/+; SmoLOF/+ (Control), Tbx18cre/+; SmoLOF/LOF (SmoLOF)	Analysis of the patency of the ureter and its junctions by ink injection into the renal pelvis
1 W-X	E18.5	3	Tbx18cre/+; SmoLOF/+ (Control), Tbx18cre/+; SmoLOF/LOF (SmoLOF)	Histological characterization of the ureter-bladder junction
1 Y-Z'	E14.5	6	Tbx18cre/+; SmoLOF/+ (Control), Tbx18cre/+; SmoLOF/LOF (SmoLOF)	Ureter explant cultures
2 A	E12.5/E14.5/E16.5	3/3/3	Tbx18cre/+; SmoLOF/+ (Control), Tbx18cre/+; SmoLOF/LOF (SmoLOF)	Histological characterization of the onset of the ureter phenotype
2 B-D	E12.5/E14.5/E16.5	3/3/3	Tbx18cre/+; SmoLOF/+ (Control), Tbx18cre/+; SmoLOF/LOF (SmoLOF)	Immunfluorescence analysis of the time course of smooth muscle differentiation
2 E-F	E12.5/E14.5/E16.5	3/3/3	Tbx18cre/+; SmoLOF/+ (Control), Tbx18cre/+; SmoLOF/LOF (SmoLOF)	Immunfluorescence analysis of the time course of urothelial differentiation
2 G	E12.5/E14.5	3/3	Tbx18cre/+; SmoLOF/+ (Control), Tbx18cre/+; SmoLOF/LOF (SmoLOF)	Analysis of cell death
2 H-I	E12.5/E14.5	3/4	Tbx18cre/+; SmoLOF/+ (Control), Tbx18cre/+; SmoLOF/LOF (SmoLOF)	Analysis of cell proliferation
3 A-B	E11.5 + 8d	6	Tbx18cre/+; R26mTmG/+ (Control), Tbx18cre/+; R26mTmG/SmoGOF (SmoGOF)	Epifluorescence analysis of explant cultures
3 C-H	E11.5 + 8d	3	Tbx18cre/+; R26mTmG/+ (Control), Tbx18cre/+; R26mTmG/SmoGOF (SmoGOF)	Histology and immunfluorescence analysis of smooth muscle differentiation
3 I-P	E11.5 + 8d	3	Tbx18cre/+; R26mTmG/+ (Control), Tbx18cre/+; R26mTmG/SmoGOF (SmoGOF)	In situ hybdization analysis of expression of fibrocyte markers
3 Q-R	E11.5 + 8d	3	Tbx18cre/+; R26mTmG/+ (Control), Tbx18cre/+; R26mTmG/SmoGOF (SmoGOF)	Immunfluorescence analysis of urothelial differentiation
4 A-B	E12.5	3	Tbx18cre/+; R26mTmG/+ (Control), Tbx18cre/+; R26mTmG/SmoGOF (SmoGOF)	Morphological characterization of urogenital systems
4 C-D	E12.5	3	Tbx18cre/+; R26mTmG/+ (Control), Tbx18cre/+; R26mTmG/SmoGOF (SmoGOF)	Histological characterization of the proximal ureter
4 E-G	E12.5	3	Tbx18cre/+; R26mTmG/+ (Control), Tbx18cre/+; R26mTmG/SmoGOF (SmoGOF)	Analysis of cell proliferation
4 H-I	E11.5 + 1d	4	Tbx18cre/+; R26mTmG/+ (Control), Tbx18cre/+; R26mTmG/SmoGOF (SmoGOF)	Analysis of cell death
4 J-K	E12.5 + 6d	6	Tbx18cre/+; R26mTmG/+ cultured with DMSO or 2 µM puromorphamine	Maintenance of ureteric mesenchymal cells by activation of HH signaling
5 C	E12.5/E14.5	3/3	Tbx18cre/+; SmoLOF/+ (Control), Tbx18cre/+; SmoLOF/LOF (SmoLOF), Tbx18cre/+; R26SmoGOF/+ (SmoGOF)	In situ hybridization analysis of expression of microarray candidate genes in the ureter
6 A-H	E14.5 + 6d	3	Tbx18+/+, Hprt:Foxf1DN/y (Control), Tbx18cre/+; Hprt:Foxf1DN/y (Foxf1DN)	Histology and immunofluorescence analysis of smooth muscle and urothelial differentiation
6 I	E12.5/E14.5	3/3	Tbx18+/+, Hprt:Foxf1DN/y (Control), Tbx18cre/+; Hprt:Foxf1DN/y (Foxf1DN)	Histology anaylsis
6 J-N	E12.5/E14.5	3/3	Tbx18+/+, Hprt:Foxf1DN/y (Control), Tbx18cre/+; Hprt:Foxf1DN/y (Foxf1DN)	Molecular analysis
6 O	E12.5/E14.5	3/3	Tbx18+/+, Hprt:Foxf1DN/y (Control), Tbx18cre/+; Hprt:Foxf1DN/y (Foxf1DN)	Analysis of cell death
6 P-Q	E12.5/E14.5	3/3	Tbx18+/+, Hprt:Foxf1DN/y (Control), Tbx18cre/+; Hprt:Foxf1DN/y (Foxf1DN)	Analysis of cell proliferation
7 A-D	E12.5 + 6d	3	Axin2+/+; Hprt:Foxf1/y (Wt), Axin2creERT2/+; Hprt:Foxf1/y with DMSO or 10 µM cyclopamine	Histology and immunofluorescence analysis of smooth muscle and urothelial differentiation
7 E-F	E12.5 + 6d	3	NMRI with DMSO or 10 µM cyclopamine and with or without 100ng/ml BMP4	Histology and immunofluorescence analysis of smooth muscle and urothelial differentiation
7 I-L	E12.5 + 6d	3	Tbx18+/+; Hprt:Foxf1DN/y (Control), Tbx18cre/+; Hprt:Foxf1DN/y with or without 100ng/ml BMP4	Histology and immunofluorescence analysis of smooth muscle and urothelial differentiation
7 M-P	E12.5 + 6d	3	Axin2+/+; Hprt:Foxf1/y (Wt), Axin2creERT2/+; Hprt:Foxf1/y with 10µg/ml NOGGIN and/or 10 µM cyclopamine, contralateral kidneys were used	Histology and immunofluorescence analysis of smooth muscle and urothelial differentiation
7Q-U	E12.5 + 6d	3	Tbx18cre/+;R26mTmG/+ with 10 µg/ml NOGGIN and 2 µM Purmorphamine, contralateral kidneys were used	Histology and immunofluorescence analysis of smooth muscle and urothelial differentiation
S1	E18.5/P40	3/3	Tbx18cre/+, R26mTmG/+	Immunofluorescence analysis of smooth muscle and urothelial differentiation
S2	E12.5 + 1d	6	NMRI with DMSO or 10 µM cyclopamine	In situ hybridization analysis of expression of *Ptch1* in ureter cultures
S3	E11.5 + 8d	4	NMRI with DMSO or 10 µM cyclopamine on day 1+2, day 2+3, day 3+4, day 4+5 or day 5+6	Whole mount immunofluorescence of smooth muscle differentiation
S4	E12.5/E14.5	3/3	Tbx18cre/+; SmoLOF/+ (Control), Tbx18cre/+; SmoLOF/LOF (SmoLOF), Tbx18+/+; R26SmoGOF/+ (Control); Tbx18cre/+; R26SmoGOF/+	In situ hybridization analysis of expression of microarray candidate genes in the ureter
S5B	E12.5/E14.5	3/3	Tbx18cre/+; SmoLOF/+ (Control), Tbx18cre/+; SmoLOF/LOF (SmoLOF), Tbx18cre/+; R26SmoGOF/+ (SmoGOF)	In situ hybridization analysis of expression of marker genes of the undifferentiated ureteric mesenchyme
S6	E12.5/E14.5	3/3	Tbx18+/+, Hprt:Foxf1DN/y (Control), Tbx18cre/+; Hprt:Foxf1DN/y (Foxf1DN)	In situ hybridization analysis of expression of marker genes of the undifferentiated ureteric mesenchyme
S7	E12.5 + 6d	4	Axin2+/+; Hprt:Foxf1/y (Wt), Axin2creERT2/+; Hprt:Foxf1/y with DMSO or 10 µM cyclopamine	In situ hybridization analysis of *Foxf1* and *Bmp4* expression
