# Supplementary material for: A SHH-FOXF1-BMP4 signaling axis regulating growth and differentiation of epithelial and mesenchymal tissues in ureter development
Source: PLoS Genet. 2017 Aug 10;13(8):e1006951. doi: 10.1371/journal.pgen.1006951 (PMC5567910; doi:10.1371/journal.pgen.1006951)
Supplement: S2 Table — Shown are two lists of transcripts with reduced and enhanced expression after an 18-h treatment of E12.5 ureter explants with 10 μM cyclopamine. Two groups each of untreated and cyclopamine-treated ureters are shown with their intensities, and the resulting fold changes in expression upon comparison. For genes with reduced intensities after cyclopamine treatment, the intensity threshold was 200 for the control; fold changes were smaller than -1.4. For genes with enhanced intensities after cyclopamine treatment the intensity threshold was 200 for the treated group; fold changes were larger than 1.4. (PDF) [file pgen.1006951.s009.pdf]

Genes with reduced expression in the ureter after 18 h treatme

Identifier	Gene Name	Intensity Control 1	Intensity Cycylopamine 1	Intensity Control 2
NM_020259	Hhip	1581	123	1326
NM_010426	Foxf1	4668	892	4757
NM_008024	Foxl1	3028	771	3306
NM_010225	Foxf2	1329	446	1105
AK147626	Ptch1	1238	344	1065
NM_016669	Crym	1573	390	1098
NM_030143	Ddit4l	402	109	345
AK136956	Hapln3	301	87	292
AK014270	Ptchd4	450	135	343
ENSMUST00000051253	ENSMUST00000051253	1748	614	1456
NM_172485	Thsd7b	246	103	302
NM_028443	Fam101a	1068	380	859
NM_016847	Avpr1a	1116	420	1224
NM_010883	Ndp	1218	474	919
NM_011125	Pltp	888	400	860
NM_010296	Gli1	5606	2329	5170
NM_053088	Ifitm5	235	110	215
NM_001039347	Kcnd3	581	266	621
NM_011915	Wif1	1026	476	1009
NM_007934	Enpep	2515	1212	2583
NM_001024139	Adamts15	1258	650	1332
NM_019521	Gas6	2225	1066	1998
NM_021342	Kcne4	300	138	233
NM_007472	Aqp1	1077	518	873
NM_001099299	Ajap1	785	403	757
NM_178642	Ano1	1172	543	1035
NM_009856	Cd83	576	267	474
NM_013703	Vldlr	4095	2082	3883
NR_033803	6030408B16Rik	6137	3162	6232
NM_018874	Pnliprp1	1068	601	914
NM_022316	Smoc1	2470	1401	2867
NR_030721	9130206I24Rik	580	316	530
NM_001085521	Tmem90b	657	329	519
NM_011580	Thbs1	2556	1422	2964
NM_008770	Cldn11	4607	2689	4651
NM_001190870	Kcne3	234	130	197
NM_025807	Slc16a9	288	166	388
NM_144945	Lgi2	611	349	505
NM_145741	Gdf10	568	346	607
ENSMUST00000055537	Gm22	237	137	218
NM_054095	Necab2	1011	613	1000
NM_172610	Mpped1	629	387	611
NM_148938	Slc1a3	1295	707	1292
TC1679329	TC1679329	214	117	262
AK017236	5330406M23Rik	193	116	253
NM_177410	Bcl2	945	555	756
NM_011825	Grem2	191	115	238

NM_030060	Batf3	1328	810	1229
NM_022435	Sp5	414	261	407
NM_001037906	Nell1	895	502	890
NM_010500	Ier5	1749	1130	1413
NM_153543	Aldh1l2	288	184	414
NM_011427	Snai1	1277	825	1393
NR_030682	2810410L24Rik	1036	686	1114

Genes with enhanced expression in the ureter after 18 h treatn

Identifier	Gene Name	Intensity Control 1	Intensity Cycylopamine 1	Intensity Control 2
NM_017474	Clca3	234	583	131
NM_008605	Mmp12	454	772	346
NM_001163032	Synpr	288	533	251
NM_133681	Tspan1	267	548	195
NM_001126490	Ism1	595	1098	705
NM_010819	Clec4d	348	628	460
NM_001204233	Spp1	2787	5073	1798
NM_008469	Krt15	1821	3247	1119
NM_001039195	Gria2	110	195	135
NM_007726	Cnr1	434	790	489
NM_033605	Dach2	982	1771	924
NM_010518	Igfbp5	16097	28770	18042
NM_013602	Mt1	7461	13221	9088
NM_021384	Rsad2	241	394	326
NM_018798	Ubqln2	820	1517	1234
NM_177597	March11	518	899	539
NM_008147	Gp49a	529	865	474
NM_008509	Lpl	3199	5070	3054
NM_011337	Ccl3	177	288	146
NM_011338	Ccl9	740	1194	629
ENSMUST00000093859	Grin3a	747	1289	734
NM_013532	Lilrb4	1014	1611	993
NR_004414	Rnu2-10	526	823	569
XR_035383	A730089K16Rik	316	496	324
NM_009807	Casp1	205	316	192
NM_053155	Clmn	1189	1855	1347

ent with cyclopamine

Intensity Cycylopamine 2	Fold change 1	Fold change 2	Average Fold change
121	-12,9	-11,0	-11,9
877	-5,2	-5,4	-5,3
835	-3,9	-4,0	-3,9
311	-3,0	-3,5	-3,3
377	-3,6	-2,8	-3,2
465	-4,0	-2,4	-3,2
130	-3,7	-2,6	-3,2
102	-3,5	-2,9	-3,2
116	-3,3	-2,9	-3,1
492	-2,8	-3,0	-2,9
113	-2,4	-2,7	-2,5
394	-2,8	-2,2	-2,5
543	-2,7	-2,3	-2,5
408	-2,6	-2,3	-2,4
346	-2,2	-2,5	-2,4
2311	-2,4	-2,2	-2,3
95	-2,1	-2,3	-2,2
286	-2,2	-2,2	-2,2
501	-2,2	-2,0	-2,1
1307	-2,1	-2,0	-2,0
664	-1,9	-2,0	-2,0
1078	-2,1	-1,9	-2,0
132	-2,2	-1,8	-2,0
473	-2,1	-1,8	-2,0
383	-1,9	-2,0	-2,0
595	-2,2	-1,7	-1,9
274	-2,2	-1,7	-1,9
2097	-2,0	-1,9	-1,9
3372	-1,9	-1,8	-1,9
460	-1,8	-2,0	-1,9
1465	-1,8	-2,0	-1,9
295	-1,8	-1,8	-1,8
331	-2,0	-1,6	-1,8
1693	-1,8	-1,8	-1,8
2561	-1,7	-1,8	-1,8
115	-1,8	-1,7	-1,8
226	-1,7	-1,7	-1,7
296	-1,7	-1,7	-1,7
337	-1,6	-1,8	-1,7
131	-1,7	-1,7	-1,7
576	-1,6	-1,7	-1,7
351	-1,6	-1,7	-1,7
842	-1,8	-1,5	-1,7
171	-1,8	-1,5	-1,7
150	-1,7	-1,7	-1,7
462	-1,7	-1,6	-1,7
143	-1,7	-1,7	-1,7

731	-1,6	-1,7	-1,7
235	-1,6	-1,7	-1,7
581	-1,8	-1,5	-1,7
868	-1,5	-1,6	-1,6
268	-1,6	-1,5	-1,6
909	-1,5	-1,5	-1,5
728	-1,5	-1,5	-1,5

nent with cyclopamine

Intensity Cycylopamine 2	Fold change 1	Fold change 2	Average Fold change
312	2,5	2,4	2,4
847	1,7	2,5	2,1
483	1,9	1,9	1,9
336	2,0	1,7	1,9
1288	1,8	1,8	1,8
855	1,8	1,9	1,8
3259	1,8	1,8	1,8
2029	1,8	1,8	1,8
245	1,8	1,8	1,8
842	1,8	1,7	1,8
1580	1,8	1,7	1,8
30658	1,8	1,7	1,7
14905	1,8	1,6	1,7
578	1,6	1,8	1,7
1915	1,8	1,6	1,7
898	1,7	1,7	1,7
835	1,6	1,8	1,7
5414	1,6	1,8	1,7
250	1,6	1,7	1,7
1046	1,6	1,7	1,6
1109	1,7	1,5	1,6
1620	1,6	1,6	1,6
906	1,6	1,6	1,6
494	1,6	1,5	1,5
299	1,5	1,6	1,5
2026	1,6	1,5	1,5
